# Recent Progress in Organic Solar Cells: A Review on Materials from Acceptor to Donor

**DOI:** 10.3390/molecules27061800

**Published:** 2022-03-10

**Authors:** Yang Li, Wei Huang, Dejiang Zhao, Lu Wang, Zhiqiang Jiao, Qingyu Huang, Peng Wang, Mengna Sun, Guangcai Yuan

**Affiliations:** BOE Technology Group Co., Ltd., Beijing 100176, China; liyang-cto@boe.com.cn (Y.L.); huangweihq@boe.com.cn (W.H.); zhaodejiang@boe.com.cn (D.Z.); wangluhq@boe.com.cn (L.W.); jiaozhiqiang@boe.com.cn (Z.J.); huangqingyu@boe.com.cn (Q.H.); wangpeng-cto@boe.com.cn (P.W.); sunmengna@boe.com.cn (M.S.)

**Keywords:** organic solar cells, acceptor, donor, ternary, tandem

## Abstract

In the last few decades, organic solar cells (OSCs) have drawn broad interest owing to their advantages such as being low cost, flexible, semitransparent, non-toxic, and ideal for roll-to-roll large-scale processing. Significant advances have been made in the field of OSCs containing high-performance active layer materials, electrodes, and interlayers, as well as novel device structures. Particularly, the innovation of active layer materials, including novel acceptors and donors, has contributed significantly to the power conversion efficiency (PCE) improvement in OSCs. In this review, high-performance acceptors, containing fullerene derivatives, small molecular, and polymeric non-fullerene acceptors (NFAs), are discussed in detail. Meanwhile, highly efficient donor materials designed for fullerene- and NFA-based OSCs are also presented. Additionally, motivated by the incessant developments of donor and acceptor materials, recent advances in the field of ternary and tandem OSCs are reviewed as well.

## 1. Introduction

Since the first silicon solar cell was invented by Bell Telephone laboratories in 1954 [1], solar cells have demonstrated great potential in utilizing renewable solar energy. After decades of development, the family of solar cells are currently composed of Si cells, inorganic thin film technologies, and emerging photovoltaics (PV). Si and various inorganic thin film solar cells have been successfully commercialized, while at the same time, the performance of emerging PV, containing OSCs, dye-sensitized solar cells (DSSCs), perovskite solar cells (PSCs), etc. also gained considerable improvement. In comparison with other PV technologies, OSCs have drawn broad interest owing to their advantages such as being low cost, flexible, semitransparent, non-toxic, and ideal for roll-to-roll large-scale processing. According to the best research-cell efficiency chart provided by the National Renewable Energy Laboratory (NREL), the PCE of OSCs has shown a rapid increase in the past few years, with the state-of-the-art OSCs yielding a certified PCE of 18.2% [2], which shows their potential for future practical applications.

**Figure 1:**
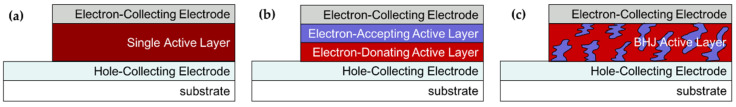
Schematic diagram of OSCs with (**a**) single active layer structure, (**b**) bilayer heterojunction structure, and (**c**) bulk heterojunction structure [10].

The first generation of OSCs was born with a single active layer, which was sandwiched between two electrodes with different work functions (Figure 1a). However, the single layer devices showed poor PCE below 0.1% for the reason of difficulty in achieving efficient dissociation of excitons (electron-hole pairs) and severe recombination of electrons and holes [3]. In 1986, a bilayer heterojunction structure (Figure 1b), containing copper phthalocyanine as donor (D) and perylene tetracarboxylic derivate as acceptor (A), was introduced by Tang [4], which was regarded as a big forward step in the field of OSCs. In this bilayer heterojunction device, copper phthalocyanine and perylene tetracarboxylic derivate stacked together as active layers, yielding a PCE of ~1%. Nevertheless, the limited D/A interface area still worked against the efficient exciton diffusion and separation, thus not yielding a high PCE in bilayer OSCs [5]. In 1995, Yu et al. [6] proposed the bulk heterojunction (BHJ) OSCs (Figure 1c), in which the donor and acceptor were mixed together to serve as the active layer. The BHJ structure presented an enhanced D/A interface and reduced the diffusion distance for exciton separation, resulting in a significant improvement in device performance. Since the invention of BHJ structure, which was considered as a breakthrough in OSCs, the PCE of BHJ OSCs has skyrocketed to over 18% [7,8,9], making it a promising PV technology.

The field of OSCs has advanced enormously in the last few decades, with frequent reports of lab-scale efficiencies of over 10% [11,12,13,14,15,16,17,18] and even 20% [19]. A large part of this progress can be attributed to the development of new light-absorbing materials. Starting from the simple homopolymers, including MDMO-PPV and P3HT, as donor and fullerene derivatives as acceptors that were popular in the early days of the field, hundreds of more complex materials for donor and acceptor have been introduced with improved optoelectric characteristics, containing light absorption, charge generation, and charge transport. Significant advances have also been made in other integral components of OSCs, including electrodes and interlayers. In addition, novel device structures such as ternary and tandem OSCs have also gained increasing attention with the great progress achieved in light-absorbing materials. In this review, we present a systematic introduction and summary of the development of donor and acceptor materials and their versatile applications in OSCs. Recent advances in the field of ternary and tandem OSCs are also discussed in this review.

## 2. D-A Materials

**Figure 2:**
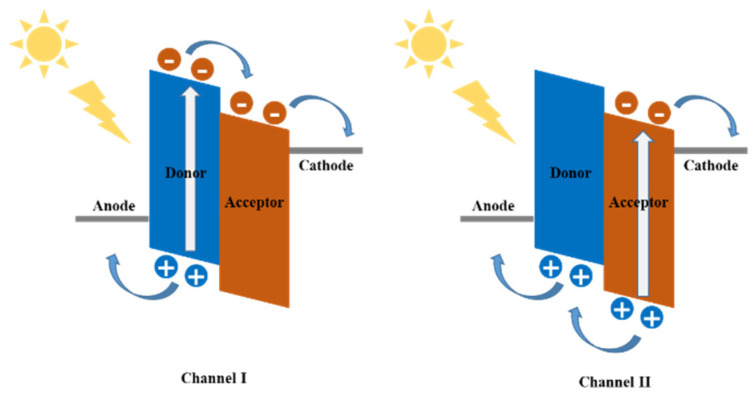
Schematic illustration of the channel I and II excitations in OSCs [20].

It is well known that in BHJ OSCs, conversion of incident photos into electric current involves four fundamental steps: (1) light absorption and exciton generation, (2) exciton diffusion to D/A interfaces, (3) exciton dissociates at D/A interfaces to form a germinate pair, (4) the charge separates and transports, which is then collected by the respective electrodes under an internal electric field [20,21]. When the active layer of BHJ OSC absorbs the photons, the excitation can occur via two kinds of paths, as shown in Figure 2. In channel I, the excitation forms in the donor, which is then followed by the electron transfer from donor to acceptor. In channel II, the excitation forms in the acceptor, which is then followed by the electron transfer from acceptor to donor. Generally, owing to different absorption abilities of acceptors, channel I dominates the photocurrent generation in fullerene-based OSCs, while in non-fullerene OSCs, both channel I and II are contributive to the generation of photocurrent.

As illustrated above, in OSCs, photoactive layer materials, including D/A materials, have always been the key factors for achieving high PCE. In general, the D/A materials in BHJ OSCs should possess the following properties: (i) matched absorption spectrum; (ii) suitable molecular energy level alignment; (iii) nanoscale phase separation; (iv) high charge carrier mobility [22].

### 2.1. Acceptor Materials

The development of acceptor materials has gone through two phases of research. Before 2015, fullerene derivatives, including PC_61_BM and PC_71_BM, were the dominant acceptors in OSCs. In 2015, Lin and co-workers reported the NFA, ITIC, which showed better absorption in the visible region, higher electron mobility, and improved D/A miscibility compared with fullerene acceptors [23]. Since then, NFAs have attracted increasing attention, and the performance of NFA-based OSCs has made remarkable progress in recent years. In the following part, we first review the acceptors in OSCs, including fullerene derivatives and NFAs.

#### 2.1.1. Fullerene Derivatives as Acceptors

In 1992, Sariciftci et al. identified for the first time that photo-induced electrons could transfer from the polymer to the fullerene cage [24]. Fullerenes are strong electron acceptors and capable of accepting up to six electrons [25]. However, owing to poor solubility and miscibility of pristine fullerenes, fullerene-based solar cells usually afforded low efficiency. In 1995, Yu and co-workers introduced the BHJ OSCs based on the soluble PC_61_BM; the blend of soluble PC_61_BM with the polymer offered an enhanced D/A interface and efficient exciton separation, thus, yielding improved photocurrent and device performance [6]. The introduction of soluble fullerene derivatives motivated the development of OSCs significantly. In 2002, Shaheen and co-workers blended PC_61_BM and MDMO-PPV together in chlorobenzene, which improved the morphology of the active layer and afforded a PCE of 2.5% [26]. Following the important breakthrough made by the use of MDMO-PPV in OSCs, research interest then shifted to polythiophenes, especially P3HT. Padinger et al. reported a PCE of 3.5% under illumination with white light at an irradiation intensity of 800 W/m^2^ based on a P3HT:PC_61_BM BHJ solar cell [27]. In 2005, Li and co-workers controlled the growth rate of P3HT:PC_61_BM, which resulted in an increased carrier mobility and charge transport, and the OSC with a PCE of 4.37% was fabricated in their work [28]. By further modification, such as thermal or vapor annealing during device fabrication, the PCE of OSCs based on P3HT:PC_61_BM has reached >5% [29].

**Figure 3:**
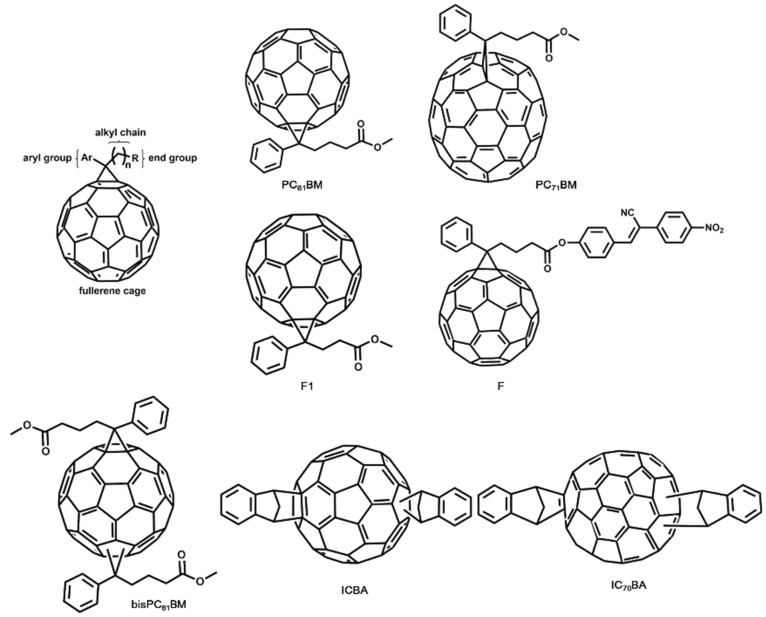
Diagram of soluble fullerene derivatives and the chemical structure of PC_61_BM, PC_71_BM, F1, F, bisPC_61_BM, ICBA, and IC_70_BA.

As Figure 3 shows, the PC_61_BM is composed of a C_60_ fullerene cage, aryl group, alkyl chain, and end group, thus there being four possible variables to improve the efficiency of fullerene-based OSCs. The first one is the aryl group modification, the second one is varying the alkyl chain length, the third one is the modification of terminal ester group, and the last is changing the fullerene cage with highly absorbing higher fullerenes. At the early stage of OSCs, PC_61_BM was used widely in the research field of solar cells because of its better performance than that of the pristine C_60_. Apart from PC_61_BM, continuous efforts have been made to increase the PCE of fullerene-based OSCs. Device parameters of OSCs based on different fullerene derivatives are summarized in Table 1.

Kim et al. prepared an aryl-substituted fullerene derivative in which the aromatic moiety of PC_61_BM was modified by replacing the monocyclic phenyl ring with bycyclic naphthalene (NC_61_BM). NC_61_BM was found to exhibit a slightly higher lowest LUMO energy level than PC_61_BM, thus leading to an enhanced Voc of 0.70 V and improved PCE of 4.09% in P3HT:NC_61_BM-based OSCs [30]. Zhao and co-workers investigated the effect of alkyl chain length of PC_61_BM on the performance of OSCs; in their work, the alkyl chain length was changed by varying the carbon atoms from 3–7, which were corresponding to F1–F5. The OSC based on P3HT:F1 system showed a PCE of 3.7%, which was slightly better than that of P3HT:PC_61_BM system (3.5%). Compared with PC_61_BM, the change in alkyl chain length affected the absorption spectra and LUMO energy level negligibly. When the F1 was mixed with P3HT, the electron mobility of the blend film gained a ~70% enhancement. In addition, P3HT:F1 film showed a better morphology than that of P3HT:PC_61_BM [31]. Besides the modification of aryl group and alkyl chain length, the way that the terminal ester group works was studied as well. Mikroyannidis et al. provided a simple and effective approach to modifying PC_61_BM, where PC_61_BM was hydrolyzed to carboxylic acid and then converted to the corresponding carbonyl chloride; correspondingly, a modified fullerene F was obtained. F showed stronger absorption, and the LUMO energy level of F was raised by 0.2 eV in comparison with that of PC_61_BM. With the assistance of enhanced properties, the OSC based on P3HT:F resulted in a PCE of 4.23%, while the P3HT:PC_61_BM device only yielded a PCE of 2.93% under the same condition. A maximum PCE of 5.25% was realized by depositing P3HT:F film from a mixture solvents and thermal annealing [32].

On the basis of the above results, the modification of PC_61_BM with aryl group, alkyl chain length, and the end group could improve the performance of OSCs to a certain extent. However, in the most cases, the modifications of PC_61_BM made no significant difference in the PCE of BHJ-OSCs. To address the drawbacks of PC_61_BM, such as poor solubility and low absorption, and to further improve the PCE of fullerene-based solar cells, fullerene derivatives with higher fullerene C_70_ were introduced into the OSCs fields.

In 2003, Wienk and co-workers synthesized PC_71_BM, which was composed of a higher fullerene cage than PC_61_BM. PC_71_BM displayed improved light absorption in the visible region, when the PC_71_BM was mixed with MDMO-PPV, the device showed 50% higher Jsc, and the overall PCE under AM1.5G amounted to 3.0% [33]. In the work of Troshin et al., a PCE of 4.1% was achieved by using P3HT:PC_71_BM as the active layer of BHJ-OSCs [34]. In order to further increase the absorption of active layers in OSCs, donor materials with a relatively low band gap were introduced into OSCs. For example, PTB7 based on thieno[3,4-b]thiophene/benzodithiophene polymers was reported by Yu’s group, and the combination of PTB7 and PC_71_BM yielded a PCE as high as 7.4%, which was the first PC_71_BM-based polymer solar cell with a PCE over 7% [35]. In 2014, Liu and co-workers reported a new donor material, PffBT4T-2OD; a device PCE of 10.4% was achieved by mixing PffBT4T-2OD with PC_71_BM, which brought the OSCs into a new stage of PCE over 10% [36].

Besides the widely used PC_61_BM and PC_71_BM, there are several other efficient fullerene derivatives acting as acceptors in OSCs, such as, bisPC_61_BM, ICBA, and so on. In 2008, Lenes et al. reported bisPC_61_BM, which was the bisadduct analogue of PC_61_BM. Compared with PC_61_BM, an increase in the LUMO energy level of ~0.1 eV was obtained in bisPC_61_BM, raising a significant enhancement of Voc in the P3HT:bisPC_61_BM solar cell. With the decline in energy loss induced by bisPC_61_BM, a PCE of 4.5% was demonstrated [37]. Similarly, He and co-workers synthesized a new soluble C_60_ derivative, indene-C60 bisadduct (ICBA), which exhibited a LUMO energy level 0.17 eV higher than that of PC_61_BM. The OSCs based on P3HT:ICBA gave a PCE of 5.44%, with a high V_OC_ of 0.84 V [38]. Zhao et al. further increased the PCE of ICBA-based OSCs to 6.48% by device optimization [39]. Based on C_70_, soluble indene-C_70_ bisadduct (IC_70_BM) was introduced by He et al., which exhibited 0.19 eV higher the LUMO energy level than that of PC_71_BM. The OSC based on P3HT with IC_70_BM as acceptor showed a Voc of 0.84 V and PCE of 5.79% [40]. By the combination of IC_70_BM with PTB7, the PCE of OSCs was further improved to 6.67% [41].

#### 2.1.2. Non-Fullerene Acceptors

Although fullerene acceptors have gained enormous attention in the field of BHJ-OSCs, fullerene-based acceptors have their own intrinsic limitations, including: (1) limited tunability of chemical structure and energy levels, (2) weak absorption ability in the visible and near-infrared (NIR) spectral ranges, (3) morphological instability, and (4) high synthetic cost, especially for the high-performance acceptor PC_71_BM [21,42]. These drawbacks have motivated the exploration of NFAs containing small molecules and polymers to further improve the performance of OSCs. Compared with fullerene acceptors, NFAs possess distinct advantages in optical absorptivity, tunability of bandgap, and frontier orbital energy levels, thus yielding higher Voc and Jsc [43]. Over the last few years, the field of NFA-based OSCs has exhibited an unprecedented progress, and the efficiency record of OSCs based on NFAs was refreshed frequently.

(1) Small molecular NFAs

**Figure 4:**
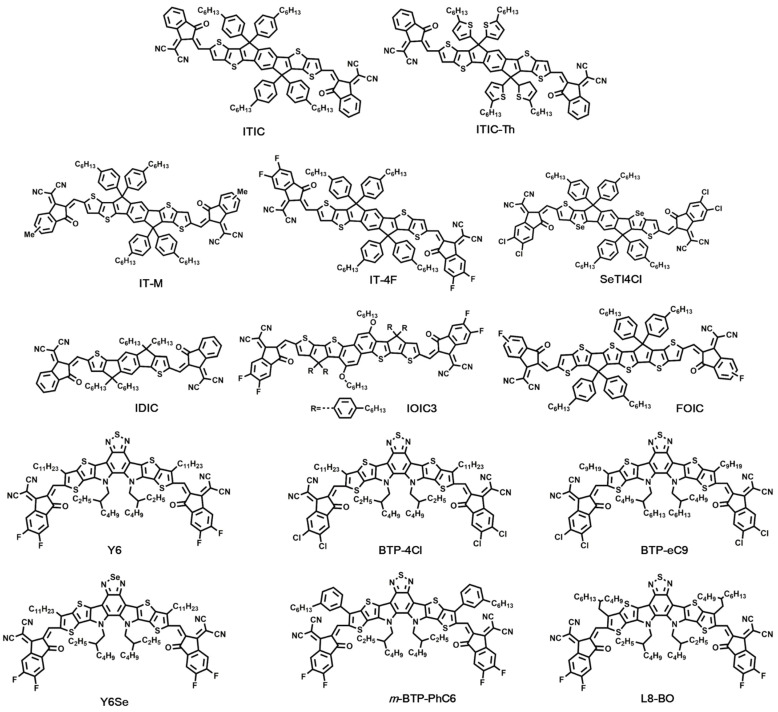
Chemical structures of representative small molecular NFAs.

Generally, there are two main strategies for obtaining efficient NFAs, including imide-based NFAs and A-D-A type NFAs. In this section, we focus on the A-D-A type NFAs, where a conjugated “push–pull” structure was applied. In A-D-A type, “A” and “D” represent the electron-withdrawing and electron-donating moieties, respectively. The combination of electron-rich and electron-deficient moieties can extend conjugation and decrease bandgap [42]. In this section, selected small molecular NFAs as shown in Figure 4 are discussed in detail. Correspondingly, device parameters of OSCs based on these efficient small molecular NFAs are listed in Table 2.

In 2015, Zhan’s group reported a novel electron acceptor, ITIC, which was based on indacenodithieno[3,2-b]thiophene (IDTT) as a core, end-capped with 2-(3-oxo-2,3-dihydroinden-1-ylidene)malononitrile (INCN) group, and with four 4-hexylphenyl groups substituted on it. In ITIC, the carbonyl and cyano groups of INCN down-shifted the LUMO energy level, the push–pull structure helped induce intramolecular charge transfer and extend absorption, and the 4-hexylphenyl groups played a role in restricting aggregation. ITIC exhibited intense absorption in the range of 500–800 nm, as well as low LUMO. OSCs fabricated using PTB7-Th as donor and ITIC as acceptor yielded a promising PCE of 6.8% [23]. The introduction of ITIC unlocked the possibility of A-D-A type molecules as alternatives of fullerene acceptors in OSCs; since then, the field of NFAs has become a hot topic.

Although ITIC showed excellent properties, the PCE was somewhat limited in Zhan’s work because the absorption of ITIC and PTB7-Th was overlapped, and the advantages of ITIC was not brought into full play. In order to obtain a matched absorption spectrum, Zhao and co-workers demonstrated a conjugated polymer, PBDB-T. The absorption spectrum of PBDB-T film was complementary with that of ITIC. Additionally, the PBDB-T:ITIC blend exhibited reduced energy loss because of better energy level alignment than those in the PBDB-T:PC_71_BM. With the device structure of indium tin oxide (ITO)/ZnO/PBDB-T:ITIC/MoO_3_/Al, a PCE of 11.21% was achieved [11]. Similarly, Xu et al. introduced the 1,3,4-thiadiazole-based wide-bandgap copolymer, PBDTS-TDZ, as donor combined with ITIC as acceptor, where a PCE of 12.80% was obtained. PBDTS-TDZ exhibited a bandgap over 2.07 eV, which could match well with the low-bandgap acceptor of ITIC; thus, the blend film of PBDTS-TDZ:ITIC showed a complementary absorption in the range of 300–800 nm. High Voc and low energy loss without sacrificing Jsc and FF were realized based on PBDTS-TDZ:ITIC devices [44].

Besides ITIC, Zhan’s group developed a series of efficient acceptors based on fused rings. By using four 2-thienyl groups as the side chains of IDTT, ITIC-Th was obtained, which showed lower energy levels and higher electron mobility than ITIC. When working with PDBT-T1, the device yielded a PCE of 9.6% [12]. By using a five-ring fused core, indacenodithiophene (IDT), they synthesized IDIC, which possessed strong absorption in the region of 500–800 nm [59], and the single-junction OSCs based on the FTAZ:IDIC blend exhibited PCEs up to 12.5% [48]. In order to further broaden the absorption of NFAs, the naphtha[1,2-b:5,6-b′]dithiophene (NDT) core with alkoxy side-chains was applied to produce IOIC3; because of the π-conjugative effect and σ-inductive effect, IOIC3 displayed narrow bandgap of 1.45 eV and remarkable absorption in 600–900 nm region. The devices based on the blend of PTB7-Th:IOIC3 yielded a high Jsc of 22.9 mA/cm^2^, thus leading to a PCE of 13.1% [49]. They also designed a fused tris(thienothiophene) building block (3TT) with strong electron-donating and molecular-packing ability. With the 3TT unit, FOIC was synthesized by using 2-(5/6-fluoro-3-oxo-2,3-dihydro-1H-inden-1-ylidene)malononitrile (FIC) as the electron-deficient end group. In FOIC, intramolecular charge transfer between 3TT and FIC was enhanced, therefore leading to a strong visible-NIR absorption in the 600–950 nm range. When the FOIC worked with PTB7-Th, a PCE of 12.0% was achieved, with Jsc as high as 24.0 mA/cm^2^ [50].

In 2016, Hou’s group successively developed IT-M by adding methyls to the end groups of ITIC. Owing to the weak electron-donating property of methyl, the LUMO energy level of IT-M was elevated by 0.04 eV compared with ITIC, demonstrating a high Voc of 0.94 V in PBDB-T:IT-M-based OSCs. Correspondingly, a PCE of 12.05% was obtained [45]. Next, they synthesized IT-4F by modifying the end group of ITIC with fluorine. The introduction of fluorine down-shifted the HOMO and LUMO energy levels without causing strong steric hindrance. Meanwhile, enhanced inter/intramolecular interactions and improved absorption were observed in IT-4F. Together with fluorinated donor material, PBDB-T-SF, the device gave a PCE of 12.97% [46]. In another of Hou’s works, a fused seven-heterocyclic core, SeT, was used as the electron-rich moiety, which showed stronger electron-donating ability than IDTT. Based on SeT, a narrow bandgap NFA, SeTIC4Cl, end-capped with dechlorinated terminal units, was successfully synthesized. SeTIC4Cl exhibited a strong NIR absorption with a bandgap of 1.44 eV, down-shifted HOMO/LUMO energy levels, and high electron mobility. Therefore, the OSCs based on SeTIC4Cl:PM6 blend films gave a best PCE of 13.32% [47].

Later, Yuan and co-workers reported a new class of NFA, Y6, in which a ladder-type electron-deficient-core-based central fused ring, dithienothiophen[3,2-b]-pyrrolobenzothiadiazole (TPBT) was employed. The conjugation along the length of the molecule in Y6 was preserved because of the fused TPBT unit, which allowed tuning the electron affinity. At the same time, the absorption and intermolecular interactions were enhanced owing to the utilization of 2-(5,6-Difluoro-3-oxo-2,3-dihydro-1H-inden-1-ylidene)malononitrile (2FIC) units. Moreover, the introduction of long alkyl side chains on the terminal of the central unit increased the solubility of Y6. The absorption onset for Y6 was located at 931 nm, with an optical bandgap of 1.33 eV. The HOMO and LUMO energy levels of Y6 were −5.65 eV and −4.10 eV, respectively. By working with the medium bandgap conjugated polymer PM6 as donor, the single-junction device based on the structure of ITO/PEDOT:PSS/PM6:Y6/PDINO/Al yielded a PCE of 15.7%, with the Jsc as high as 25.2 mA/cm^2^ [51]. With the great help of Y6, the PCE of NFA-based OSCs has been improved remarkably. For example, Tran et al. reported a facile approach for improving the PCE of Y6-based OSCs, where the MoOx with excellent electrical properties was utilized as the hole-transporting layer. Based on the PBDB-T-2F:Y6 blend film, the device displayed a best PCE of 17.1% [52]. Liu et al. developed an efficient copolymer donor material—namely, D18—which showed high hole mobility and complementary absorption with Y6. The solar cells with a structure of ITO/PEDOT:PSS/D18:Y6/PDIN/Ag were fabricated, and the best cell gave a PCE of 18.22%, with a remarkable Jsc of 27.70 mA/cm^2^ [7].

Because of the excellent photovoltaic performance, Y6 and its derivatives attracted the attention of many. Recently, Cui et al. conducted a new “Y-series” NFA, BTP-4Cl, by replacing the halogen atoms of the fluorinated Y6. The chlorinated acceptor BTP-4Cl exhibited a redshift in optical absorption and downshift of the LUMO energy level compared with Y6. By working with PBDB-TF, there was a reduced non-radiative energy loss (0.206 eV) in the devices based on BTP-4Cl, which contributed to the improved Voc. Benefiting from the improved absorption and Voc, a PCE of 16.5% was demonstrated [53]. Furthermore, to balance the processability of BTP-4Cl and device efficiency, they then prolonged the alkyl chains on the pyrrole rings to 2-bultyloctyl (BO), which helped improve the solubility. In addition, the optimization of alkyl chains on the edge of BTP-4Cl was conducted by shortening the n-undecyl (C11) to n-nonyl (C9). With the modifications of BO and C9, a new NFA—namely, BTP-eC9—was developed. BTP-eC9 possessed a suitable solubility and a more enhanced electron transport property than Y6. Correspondingly, a PCE as high as 17.8% was achieved based on PM6:BTP-eC9 blend films [54]. Zhang and co-workers introduced a high-performance acceptor, Y6Se, obtained by a facile approach of selenium substitution. Compared with the sulfur-containing Y6, Y6Se exhibited lower Urbach energy (20.4 meV), broader absorption spectra, and higher electron mobility. OSCs with the structure of ITO/PEDOT:PSS/D18:Y6Se/PNDIT-F3N-Br/Ag showed a best PCE of 17.7% [55]. Different from the research on changing the branching positions and size of the alkyl side chains of Y6, Chai et al. investigated the effect of the orientation of side chains on the properties of NFAs. The NFA molecule, m-BTP-PhC6, with optimal side-chain orientation was realized by the meta-positioned hexylphenyl group, which afforded enhanced optical absorption, intermolecular packing, and phase separation. By working with PTQ10, device efficiencies up to 17.7% were carried out [56].

Recently, Li and co-workers developed a series of NFAs by substituting the beta position of the thiophene unit on a Y6-based TPBT core with branched alkyl chains. Compared with Y6, L8-BO with 2-butyloctyl substitution exhibited a different molecular packing behavior. A more condensed molecular assembly occurred in the L8-BO molecule. Meanwhile, the L8-BO afforded more π–π packing forms than that of Y6, thus leading to multiple charge-hopping pathways and relatively strong electronic coupling. When mixed with PM6, the blend film yielded high carrier generation, low charge recombination, and balanced charge transport based on a multi-length-scale morphology. L8-BO also gave better absorption complementarity and energy alignment with PM6, relative to Y6. Therefore, a remarkable PCE as high as 18.32% was realized by utilizing the PM6:L8-BO system [57]. Afterwards, Song et al. utilized diiodomethane (DIM) as a solvent additive instead of the commonly employed 1,8-diiodooctane (DIO) in the active layer. The application of DIM reduced the energetic difference between the singlet excited state and charge transfer state in the PM6:L8-BO blend, thus causing a declined voltage loss of the devices. Subsequently, a high PCE of 18.60% was obtained for PM6:L8-BO OSCs, which is the best PCE reported for binary OSCs in the literature to date [58]. Meng et al. utilized an efficient hole transporting layer (HTL) based on Cobalt(II) acetate to fabricate the OSCs, which contained the structure of ITO/Co-based HTL/PM6:L8-BO/PNDIT-F3N/Ag. With the enhancement in work function and conductivity of HTL, the Voc, Jsc, and FF were improved simultaneously, thus affording a champion PCE of 18.77% [60].

Non-fullerene OSCs based on small molecular acceptors, such as ITIC, Y6, and L8-BO, have enabled exceptional PCEs, thus serving as a promising platform apart from fullerenes. Next, another promising approach for developing BHJ-based OSCs composed of polymeric semiconductors alone, which is called all-polymer solar cells, is further discussed.

(2) Polymeric NFAs

**Figure 5:**
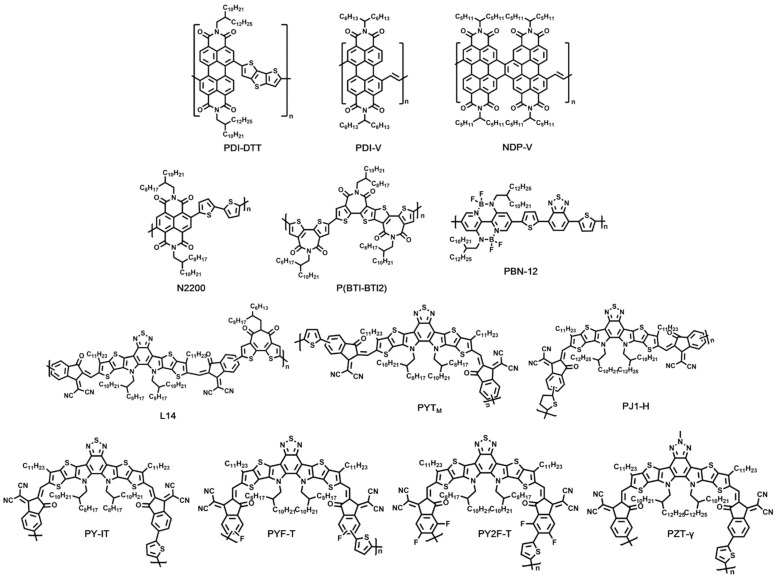
Chemical structures of representative polymeric NFAs.

All-polymer solar cells have some unique advantages, including structural flexibility, morphological stability, and outstanding mechanical properties [61]. To date, the most common building blocks for polymeric NFAs in all-polymer solar cells include perylene diimide (PDI) [62,63,64], naphthalene diimide (NDI) [13,65,66,67], bithiophene imide (BTI) [68,69], and B←N-bridged bipyridine (BN-Py) [14]. In addition, encouraged by the rapid development of small molecular NFAs, an effective approach for obtaining efficient polymer acceptors has been proposed as well, where the small molecular NFAs were utilized as A units to construct D-A copolymers [70,71,72,73,74]. Selected polymeric NFAs are displayed in Figure 5. The device parameters of OSCs based on these polymeric NFAs are listed in Table 3.

In 2007, Zhan et al. reported the application of a new polymeric acceptor based on PDI units in OSCs, which was found to possess high electron mobility. The devices based on biTV-PT:PDI-DTT blend films displayed a PCE of 1.03% at that time [62]. In spite of reasonably good electron transport property of PDI units, PDI-based polymeric acceptors generally led to low performance, which was mainly attributed to the nonplanar nature of the poly(PDI-thiophene) backbone. In order to address the above issue, Guo et al. reported a novel polymeric acceptor, PDI-V, composed of alternating PDI and vinylene units. In PDI-V, the steric hindrance near the bay region of PDI was restrained, and then the planarity of the polymer backbone as well as electron transport property were improved, thus leading to a high PCE of 7.57% in PTB7-Th:PDI-V-based OSCs [63]. Later, NDP-V was designed and synthesized by Guo and co-workers to reduce the conformational disorder in the backbone of PDI-V. The modifications led to favorable changes in the molecular packing behaviors of the acceptor and improved morphology of PTB7-Th:NDP-V blend films, thus resulting in enhanced carrier transport abilities. With this polymeric acceptor, a PCE of 8.59% was obtained for all-polymer solar cells [64].

Besides PDI, another building block widely used for polymeric NFAs is NDI, which has several advantages, such as high electron affinity, favorable charge carrier mobility, and good thermal stability. Moreover, NDI-based polymers could be prepared with a planar polymer backbone compared with PDI. NDI was first reported by Yan and co-workers in the field of organic thin-film transistor with an impressive field-effect electron mobility of 0.85 cm^2^/(V s) [76]. The NDI-based polymer, N2200, has been proved to be an efficient acceptor for all-polymer solar cells. In 2013, Mori et al. conducted the polymer:polymer BHJ solar cells utilizing PTQ1 as donor and N2200 as acceptor, the absorption of the PTQ1:N2200 blend covered the solar spectrum from visible light to 900 nm, and the LUMO–LUMO and HOMO–HOMO energy offsets were sufficient to induce free carrier generation at the heterojunction; thus, a PCE of 4.1% was realized [65]. N2200 showed weak absorption in the region from 430 to 600 nm; in order to obtain a complementary absorption for high performance all-polymer solar cells, Gao et al. introduced J51 as the donor material, which exhibited a favorable absorption from 450 to 620 nm. With the structure of ITO/PEDOT:PSS/J51:N2200/PDINO/Al, a PCE as high as 8.27% was demonstrated [66]. Fang and co-workers optimized the processing solvent and molecular weight for the production of PTzBI:N2200-based OSCs; PTzBI was used as the donor to be paired with N2200 to achieve complementary absorption. 2-Methyltetrahydrofuran (MeTHF) was explored as the solvent to improve light-harvesting ability and the morphology of the PTzBI:N2200 blend film, thus leading to a high PCE of 9.16% [67]. Later, Zhu et al. demonstrated a detailed morphological optimization; the volatile solvent MeTHF was used as well as thermal and solvent vapor annealing, leading to an optimal film morphology with improved carrier transport and reduced recombination. All-polymer solar cells with 11.76% high efficiency were then achieved by using PTzBI-Si as donor and N2200 as acceptor under printing device fabrication. [13].

Additionally, high-performance all-polymer solar cells based on BTI and BN-Py units were developed as well. For example, Shi et al. accessed a copolymer P(BTI-BTI2) based on the BTI unit, which displayed a high electron mobility of 1.23 cm^2^/(V s), evaluated by organic thin-film transistor. With the combination of PTB7-Th, the device yielded a high PCE of 8.61% based on the structure of ITO/PEDOT:PSS/PTB7-Th:P(BTI-BTI2)/LiF/Al [68]. Zhao et al. designed and synthesized an organoboron polymer, PBN-12, as the polymer acceptor based on the BN-Py unit. The absorption spectrum, energy level, electron mobility, and phase separation behavior of PBN-12 was tuned carefully; thus, OSCs based on the CD1:PBN-12 blend exhibited a favorable PCE of 10.07% [14]. Sun et al. reported a new narrow-bandgap polymer acceptor L14 by copolymerizing a dibrominated fused-ring electron acceptor with distannylated BTI. The incorporation of BTI enhanced electron mobility as well as absorption of L14, thus yielding a substantial Jsc of 20.6 m/cm^2^. Based on the structure of ITO/PEDOT:PSS/PM6:L14/PDINO/Al, a high PCE of 14.3% was achieved [69].

Recently, encouraged by the great success of small molecular NFAs, novel polymeric acceptors developed by the strategy of “small molecular NFA polymerization” were continuously proposed. In 2017, Zhang and co-workers obtained a novel polymeric acceptor, PZ1, by embedding an A-D-A building block into the polymer main chain. PZ1 afforded a narrow bandgap of 1.55 eV and high absorption coefficient, thus showing a PCE of 9.19% for all-polymer solar cells [77]. Since then, a series of high-performance polymerized small molecular NFAs were demonstrated. Wang et al. reported a π-conjugated polymeric acceptor—namely, PYT—in which a small molecular acceptor Y5-C20 was used as the key building block, and thiophene worked as the linking unit. PYT with a medium molecular weight (PYT_M_) showed a bandgap of 1.42 eV and a high absorption coefficient of 1.03 × 10^5^ cm^−1^. By working with PM6, the PYT_M_-based all-polymer solar cell exhibited a remarkable PCE of 13.44% [70]. By using a dodecyl group to replace the octyl group in PYT_M_, Jia et al. developed a polymeric acceptor, PJ1-H, all-polymer solar cell with a PCE of 14.4% that was realized by utilizing PBDB-T:PJ1-H as the active layer [72].

Among the above polymeric acceptors, brominated 1,1-dicyanomethylene-3-indanone (IC-Br) was adopted widely as a terminal unit, which exhibits a mixture of two isomers with similar polarity. In order to separate the counterparts of IC-Br, Luo and co-workers developed IC-Br (in) and IC-Br (out) from IC-Br by recrystallization of different solvents. PY-IT and PY-OT were then obtained with different polymerization sites. In the OSCs based on the PM6:PY-IT system, enhanced absorption, more balanced charge transport, and more favorable morphology with suitable domain size were achieved over that of the PM6:PY-OT system, thus affording a remarkably high PCE of 15.05% [74]. Yu and co-workers paid attention to the effects of fluorination of IC-Br on the properties of the corresponding polymeric acceptors and the all-polymer solar cells. They reported a polymeric acceptor—namely, PYF-T—by using the dihalogenated end group modified by fluorine and bromine (IC-FBr). The fluorination on the IC moiety enabled strong intramolecular charge transfer and enhanced the aggregation of the polymer acceptor, thus leading to efficient charge dissociation, rapid charge transport, and suppressed charge recombination in the PM6:PYF-T-based all-polymer solar cells. As a result, a PCE of 14.10% was demonstrated [71]. Furthermore, they synthesized PY2F-T by adopting a difluoro-monobromo end group, IC-2FBr. The performance of PY2F-T-based devices was improved significantly by the fluorination strategy, delivering a device PCE of 15.22% [75]. Recently, Fu and co-workers developed a series of PZTs as acceptors for all-polymer solar cells based on the benzotriazole-core fused-ring segment. Compared with PYT containing benzothiadiazole, PZT derivatives afforded red-shifted optical absorption and up-shifted energy levels, causing improved Jsc and Voc in the resultant devices. In addition, a regioregular PZT (PZT-γ) with higher regiospecificity was developed. All-polymer solar cells based on the PBDB-T:PZT-γ system exhibited a remarkable PCE of 15.8%, with an enhanced Jsc and a low energy loss.

The development of acceptors from fullerene derivatives to small molecular and polymeric NFAs has boosted the performance of OSCs dramatically. It is believed that numerous efforts that have been made by researchers in the field of acceptors will push further improvement of OSCs.

### 2.2. Donor Materials

Apart from highly efficient acceptors, donor materials are another crucial element in the advancement of OSCs, as illustrated above. At the beginning of BHJ-OSCs, conjugated PPV and its derivatives drew considerable attention due to their conducting and photoluminescent properties. In 1995, Yu et al. blended the MEH-PPV and PC_61_BM together as the active layer of OSCs, in which the efficiencies of charge separation and collection were enhanced remarkably [6]. Shaheen and co-workers then reported a PCE of 2.5% by exploring MDMO-PPV as donor and PC_61_BM as acceptor [26]. Afterwards, poly(thiophene)-based conjugated polymer, P3HT, has been widely used as the donor material in OSCs owing to its high carrier mobility, good solubility, and properties of crystallinity and self-assembly [78]. OSCs based on P3HT usually exhibited a PCE of 3~5% [29,39,79]. In 2010, Zhao et al. carried out detailed optimization of the OSCs based on a P3HT:ICBA blend. By optimizing the weight ratio of P3HT:ICBA and the pre-thermal annealing temperature, a PCE as high as 6.48% was reported, which was one of the highest values for the P3HT-based OSCs [39].

**Figure 6:**
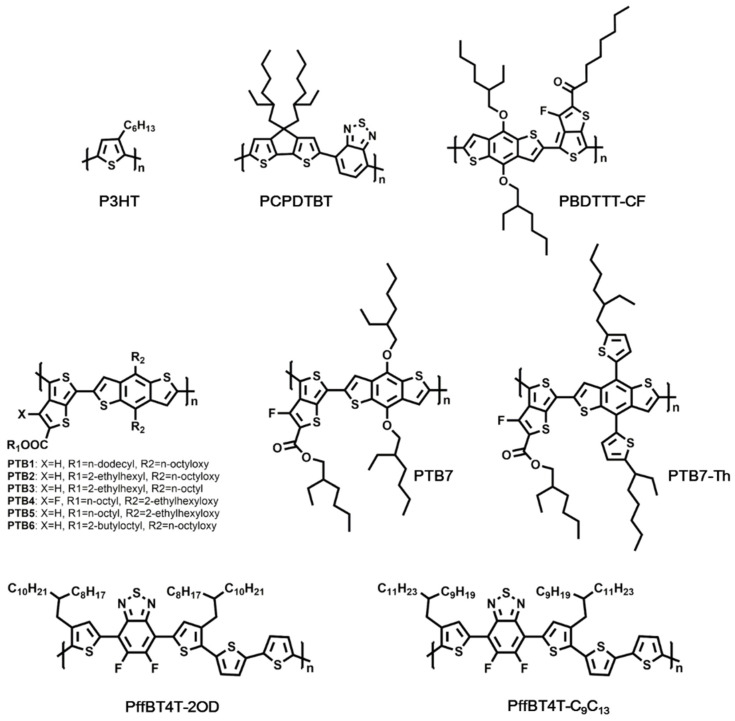
Chemical structures of representative donor materials in fullerene-based OSCs.

Before the great success of NFAs, conjugated polymers were usually designed to match fullerene acceptors for OSCs to improve the light-harvesting ability of the active layer. On the basis of optical bandgaps (Eg opt), donor materials can be divided into three major types: wide bandgap (WBG, Eg opt > 1.8 eV), medium bandgap (MBG, 1.6 eV < Eg opt ≤ 1.8 eV), and low bandgap (LBG, Eg opt ≤ 1.6 eV) [20]. The donors including MEH-PPV and P3HT all possess the wide bandgap, which yielded a relatively low Jsc in corresponding devices. In order to improve the PCE of OSCs, MBG- and LBG-donor materials were developed. Characteristics of typical donor materials (shown in Figure 6) in fullerene-based OSCs and corresponding device performance are listed in Table 4.

In 2006, Mϋhlbacher and co-workers presented a novel conjugated polymer PCPDTBT as donor in OSCs for the first time. PCPDTBT, with an optical energy gap of ~1.46 eV, exhibited absorption and photoconductive response from 300 to 850 nm. When blended with PC_71_BM, a PCE of 3.2% was demonstrated [86,87]. Later, Peet et al. incorporated a few volume of alkane dithiols as additives to modify the morphology of PCPDTBT:PC_71_BM blend film. With the optimization of heterojunction morphology, the devices afforded an average PCE of 5.5%, with Jsc = 16.2 mA/cm^2^ [80]. In 2009, a low-bandgap polymer, PBDTTT-CF, was developed by Chen et al. to modify the Voc of the devices. The absorption spectra of the PBDTTT-CF film showed an absorption edge of ~770 nm, indicating a bandgap of ~1.61 eV. OSCs based on PBDTTT-CF:PC_71_BM exhibited efficient photoconversion efficiency in the range of 400–700 nm, thus leading to a Jsc of 15.2 mA/cm^2^ in the devices. Along with the increase in Voc induced by tuning the energy level of PBDTTT-CF, a PCE as high as 7.73% was realized [81].

In 2008, Liang et al. reported a low bandgap polymer as donor material in OSCs, which was based on thieno[3,4-b]thiophene (TT) and benzodithiophene (BDT) units. The new polymer, PTB1, displayed an optical bandgap of ~1.63 eV, with the HOMO and LUMO energy levels being −4.90 and −3.20 eV, respectively. Moreover, the BDT unit made the backbone of PTB1 more rigid than P3HT, resulting in a higher mobility in PTB1. By further optimizing the weight ratio of PTB1:PC_71_BM, the OSCs finally gave an impressive PCE of 5.30% [82]. Soon afterwards, a series of PTB-based LBG polymers were designed and synthesized by Lu’s group. In order to increase the solubility and modify the LUMO/HOMO energy levels of the PTB-based polymers, different side chains such as n-octyl, 2-butyloctyl, and 2-ethylhexyloxy were explored by Lu’s group. Finally, a fluorinated polymer, PTB4, which was based on BDT substituted with branch side chain, was demonstrated, with LUMO and HOMO energy levels being −3.31 and −5.12 eV, respectively. The OSCs using PTB4:PC_61_BM blend as light-harvesting layer afforded a PCE of 5.90% [83]. In 2010, after an extensive structural optimization, Lu’s group further developed a new polymer from the PTB family, PTB7. A high PCE up to 7.40% was achieved from PTB7:PC_71_BM-based OSCs, which were the first polymer solar cells exhibiting a PCE over 7%. In PTB-based polymers, the rigid backbone led to a good hole mobility, and the side chains on the ester and BDT enabled good solubility in organic solvents and favorable miscibility with fullerides, while the incorporation of fluorine into the TT lowered the HOMO energy level. All the above advantages contributed to the high performance of PTB7-based OSCs [35].

In 2012, He et al. demonstrated highly efficient OSCs based on a PTB7:PC_71_BM blend with a certified efficiency of 9.214% using an inverted structure. Relative to conventional structures, inverted devices afford better stability due to the absence of hydrophilic PEDOT:PSS. Meanwhile, inverted OSCs can make better use of the vertical phase separation and concentration gradient in the active layer. He and co-workers used PFN to modify the work function of ITO, thus resulting in an ohmic contact at the interface for photogenerated charge carrier collection [84]. To further extend the absorption of PTB7, Chen’s group incorporated the 2-ethylhexyl-thienyl group into the BDT unit of PTB7 so that the coplanarity of the main chain was improved. The novel copolymer—namely, PTB7-Th—showed an optical bandgap of 1.58 eV, which is 0.05 eV lower than that of PTB7. Owing to the narrower bandgap, the absorption range of PTB7-Th (500–785 nm) was extended toward the longer wavelength by 25 nm compared with PBT7 (500–760 nm). Additionally, PTB7-Th exhibited a higher HOMO energy level (−5.22 eV) than PBT7 (−5.14 eV); therefore, a higher Voc was expected in PTB7-Th-based OSCs. By using the inverted device structure of ITO/ZnO-C_60_/PTB7-Th:PC_71_BM/MoO_3_/Ag, a remarkable PCE of 9.35% was achieved [85]. Later, Chen et al. reported a novel device structure by nanoimprinting the deterministic aperiodic nanostructures into PTB7-Th:PC_71_BM-based OSCs. Compared with the control device with a flat structure, the nanostructured OSCs afforded enhanced light-harvesting without sacrificing the charge transport properties, thus yielding a significant increase in photocurrent and a remarkable PCE of 10.10% [15].

In 2014, Yan’s group reported the achievement of high-performance thick-film OSCs based on a novel donor polymer, PffBT4T-2OD. In relation to PTB7-based materials systems, an optimum morphology containing highly crystalline and sufficiently pure yet reasonably small polymer domains was realized in PffBT4T-2OD-based OSCs. The 2-octyldodecyl alkyl chains sitting on quaterthiophene was the key structure that led to the polymer’s highly temperature-dependent aggregation behavior, which then allowed the controlled aggregation and strong crystallization of PffBT4T-2OD during the film cooling and drying process. In addition, PffBT4T-2OD exhibited a bandgap of 1.65 eV as well as enhanced hole mobility in the polymer:fullerene blend film. Therefore, PffBT4T-2OD enabled a high PCE of 10.8%, high FF of 75%, and thick film over 250 nm in the devices containing TC_71_BM as acceptor [36]. Based on PffBT4T-2OD, Yan’s group further developed a more environmentally friendly and higher-efficiency donor material, PffBT4T-C_9_C_13_. It was found that the alkyl chain length in the polymer made a critical difference in the blend morphology of OSCs. By increasing the length of alkyl chains, the polymer backbone orientation turned from edge-on to face-on relative to the electrodes. At the same time, longer alkyl chains were found to be detrimental to the domain size and purity. The balance of these two anti-synergistic effects led to an optimized choice of C_9_C_13_. The OSC with high PCE of 11.7% was demonstrated based on the PffBT4T-C_9_C_13_:PC_71_BM system, in which a non-halogenated solvent, 1,2,4-trimethylbenzene, was used with 1-phenylnaphthalene as an additive [16].

Generally, due to the weak absorption ability of fullerene derivatives, LBG polymers are needed to broad the light-harvesting range of the active layer of OSCs. Nevertheless, most of the donor materials in fullerene-based OSCs may not be suitable for NFA-based OSCs. As mentioned above, NFAs usually exhibit stronger light-harvesting ability than fullerene derivatives. Therefore, compared with fullerene acceptors, a variety of donor materials from WBG to LBG could be used to obtain high-performance OSCs based on NFAs.

In fact, some polymers were first utilized as donor materials in fullerene-based OSCs, then introduced to OSCs based on NFAs. For instance, PDBT-T1 was presented as a novel WBG donor material for the first time by Huo et al. In their work, PDBT-T1 showed a bandgap of 1.85 eV, along with the HOMO and LUMO energy levels being −5.36 eV and −3.43 eV. When combined with PC_71_BM, the device yielded a PCE of 9.74% [88]. In 2016, Zhan’s group combined PDBT-T1 with ITIC-Th as the active layer of OSCs. The blend film of PDBT-T1:ITIC-Th exhibited a complementary absorption as well as balanced charge transport property, thus affording a PCE as high as 9.6% [12]. Similarly, in 2012, Hou’s group developed the star material, PBDB-T, for the first time. A PCE of 6.67% was then obtained from PBDB-T:PC_61_BM-based OSCs [89], which was not that outstanding among fullerene-based OSCs. When incorporated in NFA-based OSCs, PBDB-T started to show its great potential for constructing high-performance devices. Hou’s group found that PBDB-T:ITIC showed a broader absorption and a more suitable energy level alignment than that of PBDB-T:PC_71_BM. With the combination of PBDB-T and ITIC, the device presented a PCE as high as 11.21% [11]. Recently, Yuan et al. demonstrated high-performance OSCs based on PBDB-T:Y1 blend film, where efficient charge generation and nonphase-segregated morphology were obtained. The optimal device afforded a PCE of 13.42%, with the Jsc as high as 22.44 mA/cm^2^ [90].

On the basis of PBDB-T, Hou’s group carried out various modifications to improve its optical and electrical properties as an efficient donor material. For example, the rational molecular optimization of PBDB-T via fluorination was performed. The fluorinated polymer, PBDB-T-SF, showed down-shifted HOMO/LUMO energy levels compared with its nonfluorinated counterpart. Additionally, the absorption peak of PBDB-T-SF film at 626 nm was improved clearly in relation to PBDB-T film. Attributed to the suitable energy level alignment and enhanced absorption, the devices based on ITO/ZnO/PBDB-T-SF:IT-4F/MoO_3_/Al yielded a champion PCE of 13.10% [46]. Afterwards, two conjugated polymers, PBDB-T-2F and PBDB-T-2Cl, were developed. The thiophene side groups in PBDB-T-2F were fluorinated, while those of PBDB-T-2Cl were chlorinated. Compared with PBDB-T-2F, PBDB-T-2Cl displayed similar optoelectronic and morphological properties, except for energy levels, thus leading to a PCE over 14% in the PBDB-T-2Cl-based OSCs [17]. Based on PBDB-T-2F, which was also known as PM6, a PCE up to 15.7% was carried out [51]. With incessant optimization, OSCs with a PCE approaching 18% were demonstrated based on PM6 in Hou’s group [54]. At the same time, Ma and co-workers proved that PBDB-T-2Cl, which was equal to PM7, could also afford high-performance OSCs. When combined with Y6, PM7-based devices gave a best PCE of 17.037%, with the Jsc being 25.644 mA/cm^2^ [18]. To further increase the withdrawing property of the acceptor building block of PBDB-T-based polymer, the ester-substituted thiophene (EST) unit was incorporated into a PM6 polymer; thus, a novel copolymer of T1 was presented via the still coupling reaction. T1 showed a gradually lower HOMO energy level and broader absorption than PM6. OSCs based on T1:IT-4F blend films afforded a best PCE of 15.1% owing to simultaneously improved Voc and Jsc [91].

In 2012, Li’s group developed a conjugated side-chain-isolated D-A copolymer—namely, J51. J51 was composed of the donor unit of BDT with a thiophene-conjugated side chain, thiophene π bridge, and the acceptor unit of benzotriazole (BTA) with fluorine substitution, which demonstrated well-defined absorption and high hole mobility. In the absence of NFAs, the OSCs containing J51:PC_71_BM as active layer exhibited a relatively low PCE of 6.00% [92]. Later, J51 was brought into the field of NFA-based OSCs. The combination of J51 and ITIC displayed well-matched complementary absorption with high intensity in the range from 300 to 780 nm. Additionally, the blend film of J51:ITIC owned balanced carrier mobilities, suitable energy alignment, and favorable film morphology, thus leading to a PCE up to 9.26% [93]. Apart from J51, Li’s group presented a series of donor materials based on BDT and BTA units to match well with NFAs. J52, J60, and J61 were then designed with branched alkyl, branched alkylthio, and linear alkylthio substituent on the thiophene conjugated side chain of BDT. The linear alkylthio helped J61 obtain red-shifted absorption, down-shifted HOMO energy level, and shorter π–π stacking distance when blended with ITIC. Therefore, the OSCs based on J61:ITIC with thermal annealing yielded a PCE of 9.53% [94]. With continuous modification of the thiophene side chain of BDT, Li’ group reported more efficient donor materials, J71, J81, and J91, which showed optical bandgap of 1.96 eV, 1.93eV, and 2.00 eV, respectively. Due to enhanced absorption, improved hole mobility, and declined energy loss, the OSCs based on J71, J81, and J91 yielded a best PCE of 11.63% [95,96,97].

Recently, Ding’s group reported high-performance WBG copolymers based on a fused-ring aromatic lactone (FRAL) building block. Because of the strong electron-withdrawing capability and extended molecular plane of FRAL, the polymers usually exhibit deep HOMO energy levels and high hole mobility. For example, a new copolymer D16 was presented by using 5H-dithieno[3,2-b:2′,3′-d]thiopyran-5-one (DTTP). The optical bandgap of D16 was 1.95 eV, along with its HOMO and LUMO energy levels being −5.48 and −2.83 eV, respectively. By optimizing the D/A ratio, active layer thickness, and additives, the OSCs containing the structure of ITO/PEDOT:PSS/D16:Y6/PDIN/Al gave a PCE of 16.72% [98]. Later, a more efficient copolymer, D18, was developed by Ding’ group, where a fused-ring acceptor unit, dithieno[3′,2′:3,4;2″,3″:5,6]benzo[1,2-c][1,2,5]thiadiazole (DTBT), was utilized. In relation to DTTP, DTBT owned a larger molecular plane, thus leading to a higher hole mobility of D18 than that of D16. With a wide bandgap of 1.98 eV, the blend film of D18:Y6 presented a complementary absorption. The best D18:Y6 cells yielded a PCE of 18.22%, with a Jsc as high as 27.70 mA/cm^2^. The PCE of 18.22% was the highest value achieved from single-junction OSCs to date [7]. After the great success of the D18:Y6 system, Ding’s group paired N3, which displayed better electrical properties than Y6 [99], as the acceptor material with D18. A PCE as high as 18.56% was achieved in the OSCs with a structure of ITO/PEDOT:PSS/D18:N3/PDIN/Ag [100]. Additionally, based on D18, they developed another efficient chlorinated copolymer donor material—namely, D18-Cl—which displayed slightly deeper HOMO and LUMO energy levels. OSCs based on D18-Cl:N3 blend delivered a high PCE of 18.13% [101].

**Figure 7:**
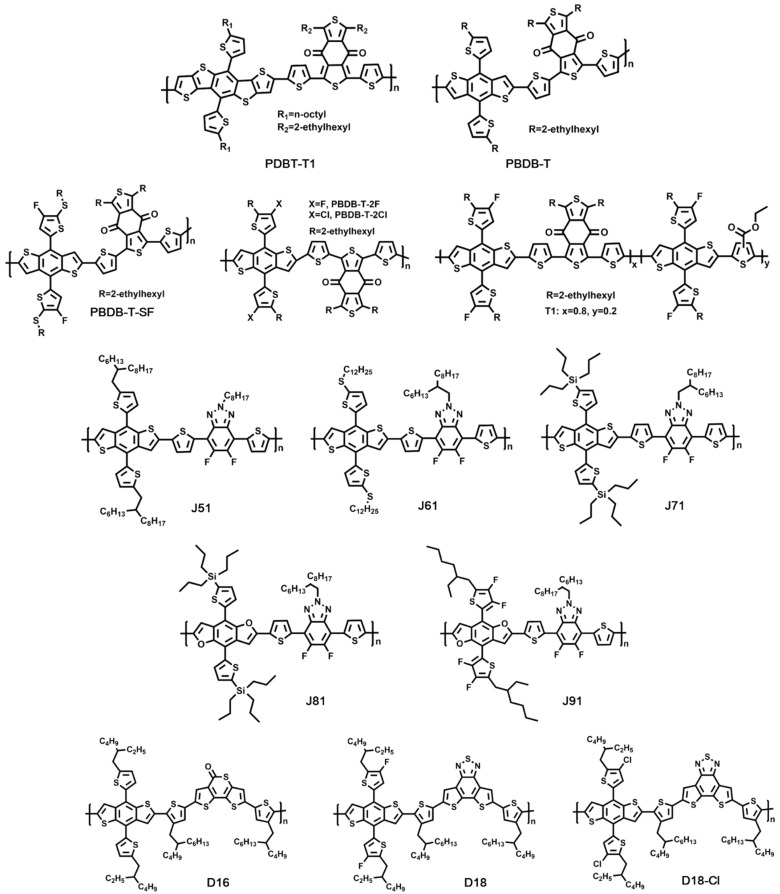
Chemical structures of representative donor materials in NFA-based OSCs.

Chemical structures of donor materials mentioned above are displayed in Figure 7. Table 5 summarizes the donor materials used in NFA-based OSCs and corresponding device parameters.

## 3. Ternary and Tandem Cells

### 3.1. Ternary OSCs

**Figure 8:**
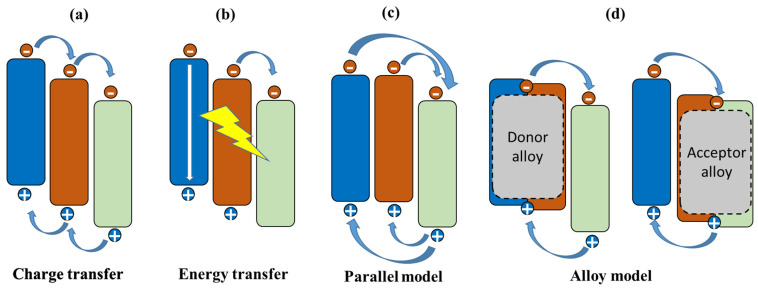
Mechanisms in ternary OSCs: (**a**) charge transfer (CT) mechanism, (**b**) energy transfer (ET) mechanism, (**c**) parallel model, and (**d**) alloy model [107].

The performance of OSCs has been improved greatly by efforts of new donor and acceptor materials. Since organic materials exhibit an absorption width of only about 0.5 eV, the binary single-junction can only take advantage of a small part of the solar radiation, which hinders the improvement in device efficiency [21]. To further improve the PCE of OSCs in order to compete with other PV technologies, ternary blend OSCs comprising either two donors and one acceptor (D1:D2:A) or one donor and two acceptors (D:A1:A2) have been developed. The ternary strategy not only could enhance the absorption coverage by using multiple materials with different bandgaps but also could preserve the processing simplicity of the binary devices [21,102]. Moreover, the incorporation of a third component also could play a crucial role in tuning the frontier molecular orbital (FMO) levels by forming homogeneous donor or acceptor phases [103,104] and modulating the active layer’s electric properties by improving the film morphology [105,106]. Finally, either the Jsc, or Voc, or FF could be promoted in ternary devices, thus boosting the PCE remarkably. Because of the above mentioned superiorities, ternary OSCs have attracted a significant amount of attention and have experienced rapid progress with a PCE of over 18.0% [8,9]. As shown in Figure 8, it is recognized that there are four fundamental mechanisms in ternary OSCs: charge transfer, energy transfer, parallel linkage, and the alloy model. The fundamental mechanisms in ternary OSCs have been reviewed in detail elsewhere [107]; here, we focus on a discussion of recent demonstrations of high-performance ternary OSCs. Ternary OSCs parameters are summarized in Table 6.

In 2009, Koppe and co-workers demonstrated a generally applicable approach for enhancing the spectroscopic response of a WBG blend of P3HT:PC_61_BM by mixing them with a LBG polymer, PCPDTBT, which was one of the earliest reported ternary OSCs. The Jsc of devices based on P3HT:PC_61_BM blend was enhanced by the addition of a proper fraction of PCPDTBT as a result of the increased NIR photo responsivity of the active layer. When 20% of PCPDTBT was added, devices based on P3HT:PCPDTBT:PC_61_BM blend yielded a PCE of 2.8%, while the P3HT:PC_61_BM-binary devices showed a PCE of 2.5% [128]. In 2013, Huang et al. utilized SQ in ternary OSCs to improve both the photo absorption range and exciton harvesting, where SQ functioned as a long-wavelength absorber owing to its high absorbance in the red and NIR spectral regions. Meanwhile, the Förster resonance energy transfer (FRET)-based system was realized by the mixture of SQ, P3HT, and PC_61_BM, thus enabling the effective use of multiple donors to obtain further enhancement of light absorption and conversion. Ternary OSCs with a P3HT:SQ:PC_61_BM blend produced a PCE of 4.51%, which was 38% higher than corresponding binary devices [108].

Although the ternary strategy is an effective approach for improving the efficiency OSCs, the PCE still remained at a relatively low level owing to the limits of donor and acceptor materials at the early stage. With the emergency of efficient LBG donor materials and NFAs, the PCE of ternary OSCs has experienced a rapid expansion.

In 2015, Lu et al. mixed PID2 as the additional donor material into PTB7-Th:PC_71_BM binary OSCs. An inspiring PCE of 9.20% for the ternary devices with 20% PID2 content was carried out. Compared with binary devices, the improved performance induced by the third component resulted from synergistic effects of enhanced light absorption, increased hole extraction ability, efficient energy transfer, better morphology, and suppressed trap-assisted recombination, thus leading to overall increases in Jsc, Voc, and FF in ternary OSCs [109]. To address the drawbacks of inferior hole mobility and poor crystallinity of PTB7-Th, Zhao et al. chose PffBT4T-2OD, which exhibited wide absorption, proper energy levels, and good crystallinity, as the third component to incorporate into a PTB7-Th:PC_71_BM binary blend. A notable PCE of 10.72% was achieved for the ternary OSCs, with 15% weight ratio of PffBT4T-2OD. The devices with 15% PffBT4T-2OD possessed proper domain size (25 nm) and enhanced domain purity; therefore, a simultaneously improved Jsc and FF was produced due to efficient charge dissociation, uplifted hole mobility, and inhibited monomolecular recombination. When the ratio of PffBT4T-2OD increased, the ternary blend maintained high domain purity but increased domain size larger than 50 nm, thus hindering charge dissociation and transport. In addition, energy transfer from PffBT4T-2OD to PTB7-Th was confirmed by transient absorption, which was helpful to the improved exciton generation [110]. Similarly, based on PTB7-Th:PC_71_BM binary blend, Du et al. used a small molecule, DIBC, to make up the weak absorption range between 500 and 600 nm of the binary films. As the concentration of DIBC increased from 0% to 30%, the absorption spectra of PTB7-Th:DIBC:PC_71_BM blend exhibited remarkable enhancement at the range of 480–570 nm. As a result, ternary OSCs with a best PCE of 12.17% were carried out, along with good tolerance to film thickness and blend ratios of DIBC [111].

Compared with fullerene acceptors, NFAs usually exhibit stronger absorption. To make the best of the complementary absorption of donors and acceptors, Zhong et al. developed highly efficient NFA-based ternary OSCs with J51 and PTB7-Th as donors and ITIC as acceptor. A best PCE of 9.70% was realized at the optimal 20% weight ratio of PTB7-Th addition. The synergistic effects of enhanced light absorption, energy transfer between J51 and PTB7-Th, improved morphology, and balanced hole/electron mobilities accounted for the improved Jsc as well as the high device efficiency [112]. Xu et al. reported ternary OSCs based on a LBG copolymer of PTB7-Th, a MBG copolymer of PBDB-T, and a WBG small molecule of SFBRCN. The ternary blend film exhibited a good complementary absorption in the range of 300–800 nm. A best PCE of 12.27% was achieved by adding 30% of PBDB-T as the third component. Apart from the enhanced absorption, charge transfer and FRET were also responsible for the improvement in the whole device performance [102]. Nian et al. carried out highly efficient ternary OSCs with PCEs up to 13.52% by adding a strongly aggregating polymer PDTfBO-TT, which owned high hole mobility, into a PBDB-T:IT-M non-fullerene blend. A proper addition of PDTfBO-TT enhanced the crystallization of PBDB-T in the ternary blend film; meanwhile, a desirable morphology was maintained, thus resulting in enhancement of charge extraction and decline in charge recombination in comparison with the binary one [113]. Song et al. utilized a small molecule donor BIT-4F-T as the third component incorporated into the PTB7-Th:IEICO-4F blend. The investigation of BIT-4F-T revealed that BIT-4F-T functioned as a processing aid in the ternary system for increasing the overall volume fraction of the highly ordered region, thus improving the charge generation and transport. BIT-4F-T also contributed to the photocurrent via hole transfer to PTB7-Th. Meanwhile, a complementary absorption was obtained by adding 10% BIT-4F-T into PTB7-Th:IEICO-4F. Finally, a 14.0% ternary OSC was performed with the combination of BIT-4F-T and interfacial engineering, along with a Jsc as high as 27.3 mA/cm^2^ [114].

Recently, high-performance ternary OSCs with PCEs over 16% were reported frequently. Xu and co-workers demonstrated highly efficient ternary OSCs by blending PNDT-ST and PBDT-ST together with Y6-T. In their work, PNDT-ST and PBDT-ST could form an “alloy model” in the ternary blend, and a continual optimization of the morphology of PBDT-ST:PNDT-ST:Y6-T system was allowed. As a result, the optimized ternary OSCs delivered a best PCE of 16.57%, with a small energy loss of 0.521 eV [115]. Xie et al. used PM6 and J71 as donors and Y6 as acceptor in the ternary OSCs, where PM6 and J71 showed complementary absorption and similar HOMO energy. By incorporating 10 wt% J71, the ternary OSCs exhibited enhanced photo-harvesting ability and declined energy loss, thus yielding a PCE as high as 16.5% [116]. Han et al. fabricated ternary OSCs by introducing PDHP-Th into a PM6:Y6 system. By working as a polymer donor guest in the ternary blend, PDHP-Th displayed a similar energy level to PM6 and regulated overaggregation of Y6. With the help of efficient charge transfer and the favorable morphology of the blend, a 16.8%-efficiency ternary PM6:PDHP-Th:Y6 device was realized [117]. An and co-workers synthesized an efficient polymer donor S3, which was then incorporated into a PM6:Y6 system to obtain ternary OSCs. In the ternary system of PM6:S3:Y6, the two donors preferred to form an “alloy mode” due to their good compatibility, leading to improved charge generation and extraction as well as complementary absorption. The optimized ternary OSCs with the structure of ITO/PEDOT:PSS/PM6:S3:Y6/PDIN/Al achieved a best PCE of 17.53% [118].

In the works illustrated above, the strategy of ternary OSCs comprising two donors and one acceptor (D1:D2:A) was utilized, which exhibited great success in improving the performance of OSCs. As the expansion of NFAs, OSCs containing one donor and two acceptors (D:A1:A2) have attracted increasing attention as well. Next, ternary OSCs of D:A1:A2 type are discussed.

Zhou et al. reported high-efficiency small-molecule ternary OSCs, where a WBG and high-crystallinity donor BTR, a LBG and moderated crystallinity acceptor NITI, and PC_71_BM were used to form a favorable cascade-energy-level alignment in the devices. The BTR:NITI:PC_71_BM ternary blend combined the advantages of both non-fullerene and fullerene acceptors, thus affording a hierarchical morphology composed of PC_71_BM transporting highways and intricate non-fullerene phase-separated small pathways. With the achievements of balanced charge transport, efficient charge extraction, reduced bimolecular recombination, and low energy loss, a best PCE of 13.63% was produced [119]. Liu et al. reported ternary OSCs with PM6 as donor and NFAs ITCPTC and MeIC as acceptors. A synergistic effect on tuning the domain size and crystallinity of the ternary blend could be realized by the proper ratio of TICPTC and MeIC. By adding less crystalline ITCPTC into the binary blend of PM6: MeIC, the morphology of the ternary blend was improved without sacrificing the electron mobility, thus resulting in an impressive PCE of 14.13% and FF of 78.2% based on the optimal blend ratio of 1:0.4:0.6 (PM6:ITCPTC:MeIC) [120]. Li et al. reported ternary OSC with two NFAs, Y6 and IDIC, with PM6 as donor material. The best PCE of 16.51% was obtained when 20% IDIC was added into the binary blend of PM6:Y6, along with overall improvements in Jsc, Voc, and FF. The increased Jsc came from the complementary absorption and hole transfer induced by IDIC. The higher LUMO energy level of IDIC than Y6 contributed to the enhanced Voc. The increased FF benefited from the reduction in trap-assisted Shockley–Read–Hall (SRH) recombination and the increase in carrier mobility [121].

Zhan and co-workers demonstrated ternary OSCs by two NFAs working in an alloy-like model. A novel NFA, BTP-M, was utilized as the third component for the PM6:Y6 binary system. Due to the same molecular backbone between Y6 and BTP-M, the Y6:BTP-M alloy-like composite was formed with median energy levels and crystallinity between Y6 and BTP-M, thus modifying energy level alignment as well as morphology of the ternary active layer to favor photocurrent generation. Based on PM6:Y6:BTP-M blend, ternary OSCs delivered a best PCE of 17.03%, with an enhanced Voc of 0.875 V and a high Jsc of 26.56 mA/cm^2^ [122]. Later, Zhu et al. reported ternary OSCs by using a new Y-series acceptor, Y18, which yielded extended optical absorption and higher voltage than Y6. When PM6, Y18, and PC_71_BM were mixed at the ratio of 1:1.5:0.2, a high PCE up to 17.11% was achieved [123].

Recently, ternary OSCs with PCE over 18% were reported by several groups, which made a great step forward in the commercialization of OSCs. Sun’s group adapted a novel NFA, BTP-F, as a third component in NFA-based ternary OSCs. BTP-F showed a same central unit with the host acceptor, BTP-eC9, but a single fluorinated end group. It was concluded that BTP-eC9 and BTP-F had the possibility of forming a well-mixed acceptor phase in the ternary blend of PM6:BTP-eC9:BTP-F, thus affording a simultaneously improved charge transport, active-layer morphology, and reduced non-radiative recombination. The OSCs based on the PM6:BTP-eC9:BTP-F system yielded a best PCE of 18.45%, which is among the highest PCE reported for single-junction OSCs so far [124]. Similarly, they incorporated another Y6-based NFA, L8-BO-F, rather than BTP-F, into the binary system of PM6:BTP-eC9. Besides the formation of a homogeneous mixed phase, energy transfer between BTP-eC9 and L8-BO-F occurred in the ternary blend of PM6:BTP-eC9:L8-BO-F. Finally, the utilization of L8-BO-F resulted in a remarkable PCE of 18.66%, with overall increases in Jsc, Voc, and FF [125]. In Ge’s group, a novel NFA, G19, was developed. By blending with high efficient binary system of D18-Cl:Y6, faster charge transfer and suitable phase were obtained in the D18-Cl:G19:Y6-based ternary OSCs, therefore demonstrating an outstanding PCE of 18.53% [126]. Lately, Ding’s group reported ternary OSCs with the structure of ITO/PEDOT:PSS/D18-Cl:N3:PC_61_BM/PDIN/Ag. With the blend ratio of 1:1.4:0.1, a champion PCE of 18.69% was achieved, and a certified PCE of 18.1% was recorded by NREL [127].

Apart from ternary OSCs, an extension of the strategy with impressive device performance could be found in use of a quaternary blend [129,130,131]. As increasing efforts have been devoted to the design and synthesis of novel efficient materials, strategies of ternary, even quaternary, could give more potential for high-performance OSCs.

### 3.2. Tandem OSCs

In general, single-junction OSCs are principally restricted in performance, where transmission loss and thermalization loss are hard to reduce; meanwhile, due to the limitation of relatively low charge mobility of organic semiconductors, the active layers are usually within a few hundred nanometers. Based on the detailed balance limit, a single-junction OSC can only convert 30% of the solar energy under 1 sun irradiance, while a tandem structure of two cells can convert 42% [132]. Therefore, to enhance the solar energy harvesting capability and further improve the PCE, tandem OSCs were carried out. In tandem OSCs, which contain two or more devices as sub-cells that are connected in series, the Voc is a sum of the Voc of each sub-cell, and the Jsc comes from the sub-cell with relatively lower Jsc, thus indicating that the balance of Jsc and Voc for each sub-cell is crucial for high efficiency in tandem OSCs. Tandem OSCs parameters discussed here are summarized in Table 7.

Liu et al. reported homo-tandem OSCs based a specially designed small molecule, SMPV1, as donor material. A solution-processed bilayer structure of polyelectrolyte (CPE) and modified PEDOT:PSS was used as interconnection layer (ICL) for the homo-tandem devices, which produced ohmic contact with negligible resistance at the interface. Thereby, the overall optical absorption was increased in the homo-tandem OSCs, thus enabling the utilization of all the photo energy in its absorption range with the help of this excellent ICL. Finally, the optimized tandem OSCs achieved a best PCE of 10.1%, with a Voc of 1.82 V [133].

You and co-workers selected WBG polymer (P3HT) combined with ICBA as front cell active materials and LBG polymer (PDTP-DFBT) with PC_61_BM as rear cell materials, which were connected by PEDOT:PSS/ZnO. P3HT and PDTP-DFBT complementarily covered the solar spectrum from 350 to 900 nm with little spectral overlap. Due to the reduced transmission loss induced by the combination of P3HT and PDTP-DFBT, the tandem OSCs showed a best PCE of 10.6%, with a Voc of 1.53 V, which was almost equal to the sum of single junction cells’ Voc [134]. To address the issue of the unbalanced Jsc in tandem OSCs, Li et al. used two small molecules with complementary absorption, DR3TSBDT and DPPEZnP-TBO, as the front and rear sub-cell donor materials in solution-processed tandem OSCs. The film of DR3TSBDT exhibited an absorption window that extended to 700 nm, while the DPPEZnP-TBO film absorbed NIR photons up to 907 nm; thus, the combination of these two materials effectively covered the entire range of the solar spectrum up to the NIR region. With careful optimization of sub-cell thickness, a best PCE of 12.70% was carried out, along with the Jsc, Voc, and FF being 12.72 mA/cm^2^, 1.60 V, and 62.4%, respectively [135].

With the rapid emergency of efficient NFAs, the utilization of NFAs in tandem OSCs was demonstrated as well. For example, Cui et al. used two NFAs with different bandgaps in tandem OSCs to broaden the photo absorption. They fabricated a PBDB-T:ITCC-M blend as front sub-cell and PBDTTT-E:IEICO as rear sub-cell; thus, small energy loss and complementary photo response spectra of the two sub-cells were obtained. By employing an ICL based on ZnO NPs and PCP-Na, the corresponding double-junction tandem OSCs demonstrated an outstanding PCE of 13.8% [136]. Similarly, Zhang et al. reported tandem OSCs based on two efficient NFAs. F-M and NOBDT with a whole absorption range from 300 to 900 nm were designed and blended with WBG polymer PBDB-T and LBG polymer PTB7-Th, respectively. After systematically optimizing the thickness of each layer, a Voc of 1.71 V, a Jsc of 11.72 mA/cm^2^, and a FF of 70% were achieved, thus affording a PCE of 14.11% [137]. In another work, Ho and co-workers reported high-performance tandem OSCs by employing HSolar/ZnO NPs as the ICL, where FTAZ:IT-M and PTB7-Th:IEICO-4F were used as the front and rear sub-cells, respectively. HSolar/ZnO NPs showed favorable wettability on the underlying sub-cell and gave better charge extraction properties; thus, a PCE of 14.7% was achieved in the tandem solar cell [138]. In addition, Qin et al. demonstrated all-non-halogenated-solvent processed tandem OSCs. Two polymer donors (PTQ10 and PTB7-Th) and two NFAs (*m*-DTC-2Cl and BTPV-4F-eC9) were used to construct sub-cells with complementary absorption. As a result, a tandem OSC with a high PCE of 16.67% was obtained, which showed potential for future massive production [139].

Liu and co-workers reported high-performance tandem OSCs, where a binary blend of PBDB-T-2F:TfIF-4FIC and ternary blend of PTB7-Th:PCDTBT:IEICO-4F were used as the front and rear sub-cells, respectively. The single-junction devices based on binary and ternary blends produced PCEs of 12.3% and 12.2%, respectively, with the devices showing broad and high external quantum efficiency (EQE) between 300 and 950 nm. Due to the efficient sub-cells and reduction in transmission loss, a high PCE that reached to 15% was achieved [140]. Later, Huang et al. used a similar strategy that contains a binary, visible absorbing front sub-cell and a ternary NIR absorbing rear sub-cell for developing tandem OSCs. In the front sub-cell, a PM6:SFT8-4F blend was used as the active layer, while a blend of PTB7-Th:BT-CIC:BEIT-4F was employed in the rear sub-cell, thus leading to a wide absorption from 350 to 1050 nm. As a result, a maximum PCE of 15.9% was recorded [141]. Similarly, Jia et al. adopted an NFA, BTPV-4F, with an optical bandgap of 1.21 eV as the rear sub-cell acceptor added into the binary blend of PTB7-Th:PC_71_BM. By working with the front sub-cell based on PM6:m-DTC-2F, the tandem OSCs yielded an outstanding PCE of 16.4%, along with the Jsc being 14.5 mA/cm^2^ [142].

Lately, Chen’s group used a tandem strategy to overcome transmission and thermalization loss and thickness constraint of single-junction OSCs. A binary blend of PBDB-T:F-M and ternary blend of PTB7-Th:O6T-4F:PC_71_BM were used as front and rear sub-cells’ active layers, respectively, which were connected by the ICL of PEDOT:PSS/ZnO. The energy levels of each layer indicated a complementary absorption of the tandem OSC. The EQE of the tandem cells showed that the front sub-cell owned high response in the range of 300 to 720 nm, while the rear sub-cell mainly absorbed photons from 720 to 1050 nm, thus resulting in a balanced Jsc of the two sub-cells. Finally, with the systematic thickness optimization of front and rear sub-cells, a record PCE of 17.36% was achieved [143].

According to simulation results demonstrated by Jeyakumar and co-workers, the tandem OSCs of PBDTS-TDZ:ITIC in the front cell with PTB7-Th:O6T-4F:PC_71_BM in the rear cell could afford a PCE of 18.6% [145], which brought OSCs into the stage with a PCE over 18% via the tandem strategy theoretically. Afterwards, Liu and co-workers delivered a remarkable PCE of tandem OSCs up to 18.71%. An efficient ICL with ZnO NPs:PEI/PEI/PEDOT:PSS structure was explored in tandem OSCs, in which PM7:TfIF-4Cl worked as the WBG active layer in the front sub-cell, and PTB7-Th:COi8DFIC:PC_71_BM served as the LBG active layer in the rear sub-cell [144]. More encouragingly, a very recent work on tandem OSCs demonstrated by Zheng et al. showed a PCE over 20% for the first time. Electron beam-evaporated TiOx and PEDOT:PSS were used as ICL, which exhibited neat interface, high conductivity, suitable energy levels, and low Schottky barrier. Correspondingly, the tandem OSC exhibited a PCE of 20.27% [19]. On the basis of the above inspiring results, the tandem strategy has been proved to show distinct advantages in achieving high-performance OSCs. With a better match of front and rear sub-cells, along with efficient ICLs, more tandem OSCs with PCE over 20% are believed to be achievable in the future.

As illustrated above, the advances in active layer materials containing donors and acceptors have made a great contribution to the improvement in PCE in the field of OSCs. Besides the material itself, other factors, including device structure [22,146,147,148], film morphology [149,150], stability [151,152,153], and so on, play important roles in the development and application of OSCs as well. For the optimization of device structure, besides the ternary and tandem structure presented in this review, interfacial modification and surface texturization are also efficient approaches for obtaining high-performance OSCs [148,154]. Limited by the short diffusion length of exciton in organic semiconductor materials [155], the issue of film morphology of the active layer is one of the crucial factors when fabricating highly efficient devices in BHJ-OSCs. Zhao et al. discussed the potential ways of demonstrating active layers with good molecular stacking, proper domain size, high domain purity, and suitable vertical phase separation. Morphology control, including the composition of bulk heterojunction blend, processing solvent, and postfilm morphology control were presented in detail [149]. With the incessant increase in PCE, the poor stability becomes a barrier to the commercial use of OSCs. Duan and co-workers summarized the factors limiting the stability of OSCs containing oxygen, water, irradiation, heating, metastable morphology, diffusion of electrodes and buffer layers materials, and mechanical stress [152]. Potential strategies for enhancing the stability of OSCs were suggested, which gave more opportunities for the research of OSCs. In addition, various processing techniques, such as green solvents [156] and slot-die coating [157], were explored to bring OSCs more chances in the future. In this review, we focused on the recent progress of active layer materials in OSCs. Besides the donor and acceptor materials, the reviews mentioned above on device structure, film morphology of active layer, and stability provide substantial supplements to this review, thus leading to a comprehensive understanding of OSCs.

## 4. Summary

In the last few decades, OSCs have made rapid research progress through the efforts on designing high-performance photovoltaic materials. The innovations in photovoltaic materials have contributed significantly to the PCE improvement of OSCs. Many new materials have been developed for OSCs that exhibit > 10% PCE and even > 18% PCE. In this review, acceptors that contain fullerene derivatives and small molecular and polymeric NFA were discussed in detail. Currently, owing to their excellent performance, the research on novel small molecular NFAs has become a hot topic in the field of OSCs, with the efficiency record refreshing frequently. Meanwhile, continuous efforts made on fullerene derivatives and polymeric NFAs provide OSCs with more potential. Afterwards, highly efficient donor materials with different bandgaps and energy levels, which were designed for fullerene- and NFA-based OSCs, were also presented. The experimental results reviewed above clearly demonstrate the importance of well-matched donors and acceptors in terms of optoelectrionic properties. Donor and acceptor materials, featuring complementary absorption profile and matching energy levels, are preferable for developing high-performance OSCs. In addition, the incessant developments of donor and acceptor have brought ternary and tandem OSCs, which afforded PCEs over 20%—a great potential that competes with other PV technologies.

## Figures and Tables

**Table 1:** Characteristics of typical fullerene acceptors and corresponding device performance.

Acceptor	Solvent	HOMO ^a^/LUMO ^b^ (eV)	Donor	J_SC_ ^c^ (mA/cm^2^)	V_OC_ ^d^ (V)	FF ^e^ (%)	PCE (%)	Refs.
PC_61_BM	Toluene or CB ^g^	-/-	MDMO-PPV	5.25	0.82	61	2.5	[26]
PC_61_BM	DCB ^h^	-/-	P3HT	8.5	0.55	60	3.5 ^f^	[27]
PC_61_BM	DCB	-/-	P3HT	10.6	0.61	67.4	4.37	[28]
NC_61_BM	DCB	-/−3.68	P3HT	9.06	0.70	64	4.09	[30]
F1	DCB	-/−3.91	P3HT	10.8	0.564	60.3	3.7	[31]
F	Chloroform	−5.90/−3.75	P3HT	10.3	0.81	63	5.25	[32]
PC_71_BM	DCB	-/-	MDMO-PPV	7.6	0.77	51	3.0	[33]
PC_71_BM	CB	-/-	P3HT	12.2	0.61	55	4.1	[34]
PC_71_BM	CB or DCB	-/-	PTB7	14.50	0.74	68.97	7.40	[35]
PC_71_BM	CB/DCB	-/-	PffBT4T-2OD	18.2	0.77	74	10.4	[36]
bisPC_61_BM	DCB	−6.1/−3.7	P3HT	9.14	0.724	68	4.5	[37]
ICBA	DCB	-/−3.74	P3HT	9.67	0.84	67	5.44	[38]
ICBA	DCB	-/−3.74	P3HT	10.61	0.84	72.7	6.48	[39]
IC_70_BA	DCB	−5.61/−3.72	P3HT	11.34	0.81	63	5.79	[40]
IC_70_BA	CB or DCB	-/−3.83	PTB7	15.4	0.79	55	6.67	[41]

^a^ HOMO: highest occupied molecular orbital, ^b^ LUMO: lowest unoccupied molecular orbital, ^c^ Jsc: short circuit current density, ^d^ Voc: open circuit voltage, ^e^ FF: fill factor, ^f^ PCE under AM 1.5 illumination at 80 mW/cm^2^, ^g^ CB: chlorobenzene, ^h^ DCB: 1,2-dichlorobenzene.

**Table 2:** Characteristics of typical small molecular NFAs and corresponding device performance.

Acceptor	HOMO/LUMO (eV)	Donor	J_SC_ (mA/cm^2^)	V_OC_ (V)	FF (%)	PCE (%)	Refs.
ITIC	−5.48/−3.83	PTB7-Th	14.21	0.81	59.1	6.80	[23]
ITIC	−5.51/−3.78	PBDB-T	16.81	0.899	74.2	11.21	[11]
ITIC	−5.48/−3.83	PBDTS-TDZ	17.78	1.10	65.4	12.80	[44]
ITIC-Th	−5.66/−3.93	PDBT-T1	16.24	0.88	67.1	9.6	[12]
IT-M	−5.58/−3.98	PBDB-T	17.44	0.94	73.5	12.05	[45]
IT-4F	−5.66/−4.14	PBDB-T-SF	20.50	0.88	71.9	12.97	[46]
SeTIC4Cl	−5.65/−4.08	PM6	22.92	0.78	75	13.32	[47]
IDIC	−5.7/−3.9	FTAZ	20.8	0.84	71.8	12.5	[48]
IOIC3	−5.38/−3.84	PTB7-Th	22.9	0.762	74.9	13.1	[49]
FOIC	−5.36/−3.92	PTB7-Th	24.0	0.743	67.1	12.0	[50]
Y6	−5.65/−4.10	PM6	25.2	0.82	76.1	15.7	[51]
Y6	−5.7/−4.1	PM6	27.43	0.845	73.8	17.1	[52]
Y6	−5.65/−4.10	D18	27.70	0.859	76.6	18.22	[7]
BTP-4Cl	−5.65/−4.02	PM6	25.4	0.867	75	16.5	[53]
BTP-eC9	−5.64/−4.05	PM6	26.2	0.839	81.1	17.8	[54]
Y6Se	−5.70/−4.15	D18	27.98	0.839	75.3	17.7	[55]
*m*-BTP-PhC6	−5.51/−3.46	PTQ10	25.3	0.883	79.3	17.7	[56]
L8-BO	−5.68/−3.90	PM6	25.72	0.87	81.5	18.32	[57]
L8-BO	-/-	PM6	26.03	0.893	80.0	18.60	[58]

**Table 3:** Characteristics of typical polymeric NFAs and corresponding device performance.

Acceptor	HOMO/LUMO (eV)	Donor	J_SC_ (mA/cm^2^)	V_OC_ (V)	FF (%)	PCE (%)	Refs.
PDI-DTT	−5.9/−3.9	biTV-PT	4.2	0.63	39	1.03	[62]
PDI-V	−5.75/−4.02	PTB7-Th	15.9	0.74	63	7.57	[63]
NDP-V	−5.94/−4.03	PTB7-Th	17.07	0.74	67	8.59	[64]
N2200	−5.9/−4.3	PTQ1	8.85	0.84	55	4.10	[65]
N2200	−5.77/−3.84	J51	14.18	0.83	70.24	8.27	[66]
N2200	-/-	PTzBI	15.17	0.849	70.36	9.16	[67]
N2200	-/-	PTzBI-Si	17.62	0.88	75.78	11.76	[13]
P(BTI-BTI2)	−5.43/−3.55	PTB7-Th	15.66	1.01	54.01	8.61	[68]
PBN-12	−5.52/−3.45	CD1	13.39	1.17	64	10.07	[14]
L14	−5.79/−4.40	PM6	20.6	0.96	72.1	14.3	[69]
PYT_M_	−5.69/−3.92	PM6	21.78	0.95	66.33	13.44	[70]
PJ1-H	−5.64/−3.82	PBDB-T	22.6	0.90	71	14.4	[72]
PY-IT	−5.68/−3.94	PM6	22.30	0.933	72.3	15.05	[74]
PYF-T	−5.67/−3.80	PM6	23.41	0.89	67.73	14.10	[71]
PY2F-T	−5.55/−3.55	PM6	24.27	0.86	72.62	15.22	[75]
PZT-γ	−5.57/−3.78	PBDB-T	24.7	0.896	71.3	15.8	[73]

**Table 4:** Characteristics of donor materials in fullerene-based OSCs and corresponding device performance.

Donor	HOMO/LUMO (eV)	Acceptor	J_SC_ (mA/cm^2^)	V_OC_ (V)	FF (%)	PCE (%)	Refs.
P3HT	-/-	ICBA	10.61	0.84	72.7	6.48	[39]
PCPDTBT	−5.3/−3.57	PC_71_BM	16.2	0.62	55	5.5	[80]
PBDTTT-CF	−5.22/−3.45	PC_71_BM	15.2	0.76	66.9	7.73	[81]
PTB1	−4.90/−3.20	PC_71_BM	15.0	0.56	63.3	5.30	[82]
PTB4	−5.12/−3.31	PC_61_BM	13.0	0.74	61.4	5.90	[83]
PTB7	−5.15/−3.31	PC_71_BM	14.50	0.74	68.97	7.40	[35]
PTB7	−5.15/−3.31	PC_71_BM	17.46	0.754	69.99	9.214	[84]
PTB7-Th	−5.22/−3.64	PC_71_BM	15.73	0.80	74.3	9.35	[85]
PTB7-Th	-/-	PC_71_BM	19.47	0.775	66.9	10.10	[15]
PffBT4T-2OD	−5.20/−3.57	TC_71_BM	18.8	0.77	75	10.8	[36]
PffBT4T-C_9_C_13_	-/-	PC_71_BM	20.2	0.788	74	11.7	[16]

**Table 5:** Characteristics of donor materials in NFA-based OSCs and corresponding device performance.

Donor	HOMO/LUMO (eV)	Acceptor	J_SC_ (mA/cm^2^)	V_OC_ (V)	FF (%)	PCE (%)	Refs.
PDBT-T1	−5.36/−3.43	ITIC-Th	16.24	0.88	67.1	9.6	[12]
PBDB-T	−5.33/−2.92	ITIC	16.81	0.899	74.2	11.21	[11]
PBDB-T	−5.39/−3.50	Y1	22.44	0.87	69.1	13.42	[90]
PBDB-T-SF	−5.40/−3.60	IT-4F	20.88	0.88	71.3	13.10	[46]
PBDB-T-2F (PM6)	−5.47/-	IT-4F	20.81	0.84	76	13.2	[17]
PM6	−5.56/−3.50	Y6	25.2	0.82	76.1	15.7	[51]
PM6	−5.47/−3.56	BTP-eC9	26.2	0.839	81.1	17.8	[54]
PBDB-T-2Cl (PM7)	−5.51/-	IT-4F	21.80	0.86	77	14.4	[17]
PM7	−5.52/−3.57	Y6	25.644	0.897	74.0	17.037	[18]
T1	−5.48/−3.63	IT-4F	21.5	0.899	78	15.1	[91]
J61	−5.32/−3.08	ITIC	17.43	0.89	61.48	9.53	[94]
J91	−5.50/−3.02	m-ITIC	18.03	0.984	65.54	11.63	[96]
D16	−5.48/−2.83	Y6	26.61	0.85	73.8	16.72	[98]
D18	−5.51/−2.77	Y6	27.70	0.859	76.6	18.22	[7]
D18	-/-	N3	27.44	0.862	78.5	18.56	[100]
D18-Cl	−5.56/−2.78	N3	27.85	0.859	75.7	18.13	[101]

**Table 6:** Photovoltaic performances of selected ternary OSCs.

Active Layer	Type	J_SC_ (mA/cm^2^)	V_OC_ (V)	FF (%)	PCE (%)	Working Mechanism	Refs.
P3HT:SQ:PC_61_BM	D1:D2:A	11.6	0.60	64.8	4.51	ET	[108]
PTB7-Th:PID2:PC_71_BM	D1:D2:A	16.68	0.78	70.8	9.20	ET	[109]
PTB7-Th:PffBT4T-2OD:PC_71_BM	D1:D2:A	19.02	0.776	72.76	10.72	ET	[110]
PTB7-Th:DIBC:PC_71_BM	D1:D2:A	20.68	0.77	74.37	12.17	ET	[111]
J51:PTB7-Th:ITIC	D1:D2:A	17.75	0.81	67.82	9.70	ET	[112]
PDBD-T:PTB7-Th:SFBRCN	D1:D2:A	17.86	0.93	73.9	12.27	ET&CT	[102]
PBDB-T:PDTfBO-TT:IT-M	D1:D2:A	18.42	0.94	77.83	13.52	ET	[113]
PTB7-Th:BIT-4F-T:IEICO-4F	D1:D2:A	27.3	0.723	70.9	14.0	CT	[114]
PBDT-ST:PNDT-ST:Y6-T	D1:D2:A	24.04	0.91	68.30	16.57	Alloy	[115]
PM6:J71:Y6	D1:D2:A	25.55	0.85	76.0	16.5	ET	[116]
PM6:PDHP-Th:Y6	D1:D2:A	26.60	0.850	71.7	16.8	CT	[117]
PM6:S3:Y6	D1:D2:A	25.86	0.86	79.17	17.53	Alloy	[118]
BTR:NITI:PC_71_BM	D:A1:A2	19.50	0.94	73.83	13.63	CT	[119]
PM6:ITCPTC:MeIC	D:A1:A2	18.42	0.981	78.2	14.13	-	[120]
PM6:IDIC:Y6	D:A1:A2	25.39	0.868	74.92	16.51	Parallel	[121]
PM6:Y6:BTP-M	D:A1:A2	26.56	0.875	73.46	17.03	Alloy	[122]
PM6:Y18:PC_71_BM	D:A1:A2	26.3	0.84	77.4	17.11	-	[123]
PM6:BTP-eC9:BTP-F	D:A1:A2	26.99	0.858	79.7	18.45	Alloy	[124]
PM6:BTP-eC9:L8-BO-F	D:A1:A2	27.35	0.853	80.00	18.66	ET and Alloy	[125]
D18-Cl:G19:Y6	D:A1:A2	27.36	0.871	77.72	18.53	CT	[126]
D18-Cl:N3:PC_61_BM	D:A1:A2	28.22	0.849	78.0	18.69	-	[127]

**Table 7:** Photovoltaic performance of the tandem OSCs.

Front Cell	Rear Cell	ICL	J_SC_ (mA/cm^2^)	V_OC_ (V)	FF (%)	PCE (%)	Refs.
SMPV1:PC_71_BM	SMPV1:PC_71_BM	CPE/M-PEDOT:PSS	7.7	1.82	72	10.1	[133]
P3HT:ICBA	PDTP-DFBT:PC_61_BM	PEDOT:PSS/ZnO	10.1	1.53	68.5	10.6	[134]
DR3TSBDT:PC_71_BM	DPPEZnP-TBO:PC_61_BM	ZnO NPs/n-PEDOT:PSS	12.72	1.60	62.4	12.70	[135]
PBDB-T:ITCC-M	PBDTTT-E:IEICO	ZnO NPs/PCP-Na	12.0	1.80	63.9	13.8	[136]
PBDB-T:F-M	PTB7-Th:NOBDT	ZnO NPs/n-PEDOT:PSS	11.72	1.71	70	14.11	[137]
FTZA:IT-M	PTB7-Th:IEICO-4F	HSolar/ZnO NPs	14.6	1.64	67	16.1	[138]
PTQ10:m-DTC-2Cl	PTB7-Th:BTPV-4F-eC9	MoOx/M-PEDOT:PSS/ZnO NPs	14.65	1.621	70.20	16.67	[139]
PM6:TfIF-4FIC	PTB7-Th:PCDTBT:IEICO-4F	PF3N-2TNDI/Ag/PEDOT:PSS	13.6	1.60	69	15.0	[140]
PM6:SFT8-4F	PTB7-Th:BT-CIC:BEIT-4F	MoO_3_/PEDOT:PSS/ZnO NPs	14.1	1.66	68	15.9	[141]
PM6:m-DTC-2F	PTB7-Th:BTPV-4F:PC_71_BM	MoO_3_/Mix-PEDOT:PSS/ZnO NPs	14.5	1.65	68.5	16.4	[142]
PBDB-T:F-M	PTB7-Th:O6T-4F:PC_71_BM	PEDOT:PSS/ZnO NPs	14.35	1.642	73.7	17.36	[143]
PM7:TfIF-4Cl	PTB7-Th:CO_i_8DFIC:PC_71_BM	ZnO NPs:PEI/PEI/PEDOT:PSS	14.59	1.64	78	18.71	[144]
PM6:GS-ISO	PM6:BTP-eC9	e-TiO_1.76_/PEDOT:PSS	13.14	2.01	76.75	20.27	[19]

## Data Availability

Not applicable.

## References

[1] Chapin D.M., Fuller C.S., Pearson G.L. (1954). A new silicon p-n junction photocell for converting solar radiation into electrical power. J. Appl. Phys..

[2] Best Research Cell Efficiencies provided by the National Renewable Energy Laboratory (NREL). https://www.nrel.gov/pv/cell-efficiency.html.

[3] Marks R.N., Halls J.J.M., Bradley D.D.C., Friend R.H., Holmes A.B. (1994). The photovoltaic response in poly(p-phenylene vinylene) thin-film devices. J. Phys. Condens. Matter.

[4] Tang C.W. (1986). Two-layer organic photovoltaic cell. Appl. Phys. Lett..

[5] Knupfer M. (2003). Exciton binding energies in organic semiconductors. Appl. Phys. A.

[6] Yu G., Gao J., Hummelen J.C., Wudl F., Heeger A.J. (1995). Polymer photovoltaic cells: Enhanced efficiencies via a network of internal donor-acceptor heterojunctions. Science.

[7] Liu Q., Jiang Y., Jin K., Qin J., Xu J., Li W., Xiong J., Liu J., Xiao Z., Sun K. (2020). 18% Efficiency organic solar cells. Sci. Bull..

[8] Lin Y., Firdaus Y., Isikgor F.H., Nugraha M.I., Yengel E., Harrison G.T., Hallani R., El-Labban A., Faber H., Ma C. (2020). Self-assembled monolayer enables hole transport layer-free organic solar cells with 18% efficiency and improved operational stability. ACS Energy Lett..

[9] Lin Y., Nugraha M.I., Firdaus Y., Scaccabarozzi A.D., Aniés F., Emwas A.-H., Yengel E., Zheng X., Liu J., Wahyudi W. (2020). A simple n-dopant derived from diquat boosts the efficiency of organic solar cells to 18.3%. ACS Energy Lett..

[10] Kim H., Nam S., Jeong J., Lee S., Seo J., Han H., Kim Y. (2014). Organic solar cells based on conjugated polymers: History and recent advances. Korean J. Chem. Eng..

[11] Zhao W., Qian D., Zhang S., Li S., Inganäs O., Gao F., Hou J. (2016). Fullerene-free polymer solar cells with over 11% efficiency and excellent thermal stability. Adv. Mater..

[12] Lin Y., Zhao F., He Q., Huo L., Wu Y., Parker T.C., Ma W., Sun Y., Wang C., Zhu D. (2016). High-performance electron acceptor with thienyl side chains for organic photovoltaics. J. Am. Chem. Soc..

[13] Zhu L., Zhong W., Qiu C., Lyu B., Zhou Z., Zhang M., Song J., Xu J., Wang J., Ali J. (2019). Aggregation-induced multilength scaled morphology enabling 11.76% efficiency in all-polymer solar cells using printing fabrication. Adv. Mater..

[14] Zhao R., Wang N., Yu Y., Liu J. (2020). Organoboron polymer for 10% efficiency all-polymer solar cells. Chem. Mater..

[15] Chen J.-D., Cui C., Li Y.-Q., Zhou L., Ou Q.-D., Li C., Li Y., Tang J.-X. (2015). Single-junction polymer solar cells exceeding 10% power conversion efficiency. Adv. Mater..

[16] Zhao J., Li Y., Yang G., Jiang K., Lin H., Ade H., Ma W., Yan H. (2016). Efficient organic solar cells processed from hydrocarbon solvents. Nat. Energy.

[17] Zhang S., Qin Y., Zhu J., Hou J. (2018). Over 14% efficiency in polymer solar cells enabled by a chlorinated polymer donor. Adv. Mater..

[18] Ma R., Liu T., Luo Z., Guo Q., Xiao Y., Chen Y., Li X., Luo S., Lu X., Zhang M. (2020). Improving open-circuit voltage by a chlorinated polymer donor endows binary organic solar cells efficiencies over 17%. Sci. China Chem..

[19] Zheng Z., Wang J., Bi P., Ren J., Wang Y., Yang Y., Liu X., Zhang S., Hou J. (2022). Tandem organic solar cell with 20.2% efficiency. Joule.

[20] Xu X., Zhang G., Li Y., Peng Q. (2019). The recent progress of wide bandgap donor polymers towards non-fullerene organic solar cells. Chin. Chem. Lett..

[21] Dey S. (2019). Recent progress in molecular design of fused ring electron acceptors for organic solar cells. Small.

[22] Xue R., Zhang J., Li Y., Li Y. (2018). Organic solar cell materials toward commercialization. Small.

[23] Lin Y., Wang J., Zhang Z.-G., Bai H., Li Y., Zhu D., Zhan X. (2015). An electron acceptor challenging fullerenes for efficient polymer solar cells. Adv. Mater..

[24] Sariciftci N.S., Smilowitz L., Heeger A.J., Wudl F. (1992). Photoinduced electron transfer from a conducting polymer to buckminsterfullerene. Science.

[25] Seyler H., Wong W.W.H., Jones D.J., Holmes A.B. (2011). Continuous flow synthesis of fullerene derivatives. J. Org. Chem..

[26] Shaheen S.E., Brabec C.J., Sariciftci N.S., Padinger F., Fromherz T., Hummelen J.C. (2001). 2.5% efficient organic plastic solar cells. Appl. Phys. Lett..

[27] Padinger F., Rittberger R.S., Sariciftci N.S. (2003). Effects of postproduction treatment on plastic solar cells. Adv. Funct. Mater..

[28] Li G., Shrotriya V., Huang J., Yao Y., Moriarty T., Emery K., Yang Y. (2005). High-efficiency solution processable polymer photovoltaic cells by self-organization of polymer blends. Nat. Mater..

[29] Ma W., Yang C., Gong X., Lee K., Heeger A.J. (2005). Thermally stable, efficient polymer solar cells with nanoscale control of the interpenetrating network morphology. Adv. Funct. Mater..

[30] Kim H.U., Kim J.-H., Kang H., Grimsdale A.C., Kim B.J., Yoon S.C., Hwang D.-H. (2014). Naphthalene-, anthracene-, and pyrene-substituted fullerene derivatives as electron acceptors in polymer-based solar cells. ACS Appl. Mater. Interfaces.

[31] Zhao G., He Y., Xu Z., Hou J., Zhang M., Min J., Chen H.-Y., Ye M., Hong Z., Yang Y. (2010). Effect of carbon chain length in the substituent of PCBM-like molecules on their photovoltaic properties. Adv. Funct. Mater..

[32] Mikroyannidis J.A., Kabanakis A.N., Sharma S.S., Sharma G.D. (2011). A Simple and effective modification of PCBM for use as an electron acceptor in efficient bulk heterojunction solar cells. Adv. Funct. Mater..

[33] Wienk M.M., Kroon J.M., Verhees W.J., Knol J., Hummelen J.C., van Hal P.A., Janssen R.A. (2003). Efficient methano[70]fullerene/MDMO-PPV bulk heterojunction photovoltaic cells. Angew. Chem. Int. Ed. Engl..

[34] Troshin P.A., Hoppe H., Renz J., Egginger M., Mayorova J.Y., Goryachev A.E., Peregudov A.S., Lyubovskaya R.N., Gobsch G., Sariciftci N.S. (2009). Material solubility-photovoltaic performance relationship in the design of novel fullerene derivatives for bulk heterojunction solar cells. Adv. Funct. Mater..

[35] Liang Y., Xu Z., Xia J., Tsai S.T., Wu Y., Li G., Ray C., Yu L. (2010). For the bright future-bulk heterojunction polymer solar cells with power conversion efficiency of 7.4%. Adv. Mater..

[36] Liu Y., Zhao J., Li Z., Mu C., Ma W., Hu H., Jiang K., Lin H., Ade H., Yan H. (2014). Aggregation and morphology control enables multiple cases of high-efficiency polymer solar cells. Nat. Commun..

[37] Lenes M., Wetzelaer G.-J.A.H., Kooistra F.B., Veenstra S.C., Hummelen J.C., Blom P.W.M. (2008). Fullerene bisadducts for enhanced open-circuit voltages and efficiencies in polymer solar cells. Adv. Mater..

[38] He Y., Chen H.-Y., Hou J., Li Y. (2010). Indene−C60 bisadduct: A new acceptor for high-performance polymer solar cells. J. Am. Chem. Soc..

[39] Zhao G., He Y., Li Y. (2010). 6.5% efficiency of polymer solar cells based on poly(3-hexylthiophene) and indene-C60 bisadduct by device optimization. Adv. Mater..

[40] He Y., Zhao G., Peng B., Li Y. (2010). High-yield synthesis and electrochemical and photovoltaic properties of indene-C70 bisadduct. Adv. Funct. Mater..

[41] He Y., Shao M., Xiao K., Smith S.C., Hong K. (2013). High-performance polymer photovoltaics based on rationally designed fullerene acceptors. Sol. Energy Mater. Sol. Cells.

[42] Zhang G., Zhao J., Chow P.C.Y., Jiang K., Zhang J., Zhu Z., Zhang J., Huang F., Yan H. (2018). Nonfullerene acceptor molecules for bulk heterojunction organic solar cells. Chem. Rev..

[43] Fu H., Wang Z., Sun Y. (2019). Polymer donors for high-performance non-fullerene organic solar cells. Angew. Chem. Int. Ed..

[44] Xu X., Yu T., Bi Z., Ma W., Li Y., Peng Q. (2018). Realizing over 13% efficiency in green-solvent-processed nonfullerene organic solar cells enabled by 1,3,4-thiadiazole-based wide-bandgap copolymers. Adv. Mater..

[45] Li S., Ye L., Zhao W., Zhang S., Mukherjee S., Ade H., Hou J. (2016). Energy-level modulation of small-molecule electron acceptors to achieve over 12% efficiency in polymer solar cells. Adv. Mater..

[46] Zhao W., Li S., Yao H., Zhang S., Zhang Y., Yang B., Hou J. (2017). Molecular optimization enables over 13% efficiency in organic solar cells. J. Am. Chem. Soc..

[47] Wang J.-L., Liu K.-K., Hong L., Ge G.-Y., Zhang C., Hou J. (2018). Selenopheno[3,2-b]thiophene-based narrow-bandgap nonfullerene acceptor enabling 13.3% efficiency for organic solar cells with thickness-insensitive feature. ACS Energy Lett..

[48] Lin Y., Zhao F., Prasad S.K.K., Chen J.-D., Cai W., Zhang Q., Chen K., Wu Y., Ma W., Gao F. (2018). Balanced partnership between donor and acceptor components in nonfullerene organic solar cells with >12% efficiency. Adv. Mater..

[49] Zhu J., Xiao Y., Wang J., Liu K., Jiang H., Lin Y., Lu X., Zhan X. (2018). Alkoxy-induced near-infrared sensitive electron acceptor for high-performance organic solar cells. Chem. Mater..

[50] Li T., Dai S., Ke Z., Yang L., Wang J., Yan C., Ma W., Zhan X. (2018). Fused tris(thienothiophene)-based electron acceptor with strong near-infrared absorption for high-performance as-cast solar cells. Adv. Mater..

[51] Yuan J., Zhang Y., Zhou L., Zhang G., Yip H.-L., Lau T.-K., Lu X., Zhu C., Peng H., Johnson P.A. (2019). Single-junction organic solar cell with over 15% efficiency using fused-ring acceptor with electron-deficient core. Joule.

[52] Tran H.N., Park S., Wibowo F.T.A., Krishna N.V., Kang J.H., Seo J.H., Nguyen-Phu H., Jang S.-Y., Cho S. (2020). 17% Non-fullerene organic solar cells with annealing-free aqueous MoOx. Adv. Sci..

[53] Cui Y., Yao H., Zhang J., Zhang T., Wang Y., Hong L., Xian K., Xu B., Zhang S., Peng J. (2019). Over 16% efficiency organic photovoltaic cells enabled by a chlorinated acceptor with increased open-circuit voltages. Nat. Commun..

[54] Cui Y., Yao H., Zhang J., Xian K., Zhang T., Hong L., Wang Y., Xu Y., Ma K., An C. (2020). Single-junction organic photovoltaic cells with approaching 18% efficiency. Adv. Mater..

[55] Zhang Z., Li Y., Cai G., Zhang Y., Lu X., Lin Y. (2020). Selenium heterocyclic electron acceptor with small urbach energy for as-cast high-performance organic solar cells. J. Am. Chem. Soc..

[56] Chai G., Chang Y., Zhang J., Xu X., Yu L., Zou X., Li X., Chen Y., Luo S., Liu B. (2021). Fine-tuning of side-chain orientations on nonfullerene acceptors enables organic solar cells with 17.7% efficiency. Energy Environ. Sci..

[57] Li C., Zhou J., Song J., Xu J., Zhang H., Zhang X., Guo J., Zhu L., Wei D., Han G. (2021). Non-fullerene acceptors with branched side chains and improved molecular packing to exceed 18% efficiency in organic solar cells. Nat. Energy.

[58] Song J., Zhu L., Li C., Xu J., Wu H., Zhang X., Zhang Y., Tang Z., Liu F., Sun Y. (2021). High-efficiency organic solar cells with low voltage loss induced by solvent additive strategy. Matter.

[59] Lin Y., He Q., Zhao F., Huo L., Mai J., Lu X., Su C.-J., Li T., Wang J., Zhu J. (2016). A facile planar fused-ring electron acceptor for as-cast polymer solar cells with 8.71% efficiency. J. Am. Chem. Soc..

[60] Meng H., Liao C., Deng M., Xu X., Yu L., Peng Q. (2021). 18.77 % Efficiency organic solar cells promoted by aqueous solution processed cobalt(II) acetate hole transporting layer. Angew. Chem. Int. Ed..

[61] Genene Z., Mammo W., Wang E., Andersson M.R. (2019). Recent advances in n-type polymers for all-polymer solar cells. Adv. Mater..

[62] Zhan X., Tan Z.A., Domercq B., An Z., Zhang X., Barlow S., Li Y., Zhu D., Kippelen B., Marder S.R. (2007). A high-mobility electron-transport polymer with broad absorption and its use in field-effect transistors and all-polymer solar cells. J. Am. Chem. Soc..

[63] Guo Y., Li Y., Awartani O., Zhao J., Han H., Ade H., Zhao D., Yan H. (2016). A vinylene-bridged perylenediimide-based polymeric acceptor enabling efficient all-polymer solar cells processed under ambient conditions. Adv. Mater..

[64] Guo Y., Li Y., Awartani O., Han H., Zhao J., Ade H., Yan H., Zhao D. (2017). Improved performance of all-polymer solar cells enabled by naphthodiperylenetetraimide-based polymer acceptor. Adv. Mater..

[65] Mori D., Benten H., Okada I., Ohkita H., Ito S. (2014). Low-bandgap donor/acceptor polymer blend solar cells with efficiency exceeding 4%. Adv. Energy Mater..

[66] Gao L., Zhang Z.-G., Xue L., Min J., Zhang J., Wei Z., Li Y. (2016). All-polymer solar cells based on absorption-complementary polymer donor and acceptor with high power conversion efficiency of 8.27%. Adv. Mater..

[67] Fan B., Ying L., Wang Z., He B., Jiang X.-F., Huang F., Cao Y. (2017). Optimisation of processing solvent and molecular weight for the production of green-solvent-processed all-polymer solar cells with a power conversion efficiency over 9%. Energy Environ. Sci..

[68] Shi Y., Guo H., Huang J., Zhang X., Wu Z., Yang K., Zhang Y., Feng K., Woo H.Y., Ortiz R. (2020). Distannylated bithiophene imide: Enabling high-performance n-type polymer semiconductors with an acceptor-acceptor backbone. Angew. Chem..

[69] Sun H., Yu H., Shi Y., Yu J., Peng Z., Zhang X., Liu B., Wang J., Singh R., Lee J. (2020). A narrow-bandgap n-type polymer with an acceptor–acceptor backbone enabling efficient all-polymer solar cells. Adv. Mater..

[70] Wang W., Wu Q., Sun R., Guo J., Wu Y., Shi M., Yang W., Li H., Min J. (2020). Controlling molecular mass of low-band-gap polymer acceptors for high-performance all-polymer solar cells. Joule.

[71] Yu H., Qi Z., Yu J., Xiao Y., Sun R., Luo Z., Cheung A.M.H., Zhang J., Sun H., Zhou W. (2021). Fluorinated end group enables high-performance all-polymer solar cells with near-infrared absorption and enhanced device efficiency over 14%. Adv. Energy Mater..

[72] Jia T., Zhang J., Zhong W., Liang Y., Zhang K., Dong S., Ying L., Liu F., Wang X., Huang F. (2020). 14.4% efficiency all-polymer solar cell with broad absorption and low energy loss enabled by a novel polymer acceptor. Nano Energy.

[73] Fu H., Li Y., Yu J., Wu Z., Fan Q., Lin F., Woo H.Y., Gao F., Zhu Z., Jen A.K.Y. (2021). High efficiency (15.8%) all-polymer solar cells enabled by a regioregular narrow bandgap polymer acceptor. J. Am. Chem. Soc..

[74] Luo Z., Liu T., Ma R., Xiao Y., Zhan L., Zhang G., Sun H., Ni F., Chai G., Wang J. (2020). Precisely controlling the position of bromine on the end group enables well-regular polymer acceptors for all-polymer solar cells with efficiencies over 15%. Adv. Mater..

[75] Yu H., Luo S., Sun R., Angunawela I., Qi Z., Peng Z., Zhou W., Han H., Wei R., Pan M. (2021). A difluoro-monobromo end group enables high-performance polymer acceptor and efficient all-polymer solar cells processable with green solvent under ambient condition. Adv. Funct. Mater..

[76] Yan H., Chen Z., Zheng Y., Newman C., Quinn J.R., Dötz F., Kastler M., Facchetti A. (2009). A high-mobility electron-transporting polymer for printed transistors. Nature.

[77] Zhang Z.-G., Yang Y., Yao J., Xue L., Chen S., Li X., Morrison W., Yang C., Li Y. (2017). Constructing a strongly absorbing low-bandgap polymer acceptor for high-performance all-polymer solar cells. Angew. Chem. Int. Ed..

[78] Hou W., Xiao Y., Han G., Lin J.-Y. (2019). The applications of polymers in solar cells: A review. Polymers.

[79] Tan Z.A., Li S., Wang F., Qian D., Lin J., Hou J., Li Y. (2014). High performance polymer solar cells with as-prepared zirconium acetylacetonate film as cathode buffer layer. Sci. Rep..

[80] Peet J., Kim J.Y., Coates N.E., Ma W.L., Moses D., Heeger A.J., Bazan G.C. (2007). Efficiency enhancement in low-bandgap polymer solar cells by processing with alkane dithiols. Nat. Mater..

[81] Chen H.-Y., Hou J., Zhang S., Liang Y., Yang G., Yang Y., Yu L., Wu Y., Li G. (2009). Polymer solar cells with enhanced open-circuit voltage and efficiency. Nat. Photonics.

[82] Liang Y., Wu Y., Feng D., Tsai S.-T., Son H.-J., Li G., Yu L. (2009). Development of new semiconducting polymers for high performance solar cells. J. Am. Chem. Soc..

[83] Liang Y., Feng D., Wu Y., Tsai S.-T., Li G., Ray C., Yu L. (2009). Highly efficient solar cell polymers developed via fine-tuning of structural and electronic properties. J. Am. Chem. Soc..

[84] He Z., Zhong C., Su S., Xu M., Wu H., Cao Y. (2012). Enhanced power-conversion efficiency in polymer solar cells using an inverted device structure. Nat. Photonics.

[85] Liao S.H., Jhuo H.J., Cheng Y.S., Chen S.A. (2013). Fullerene derivative-doped zinc oxide nanofilm as the cathode of inverted polymer solar cells with low-bandgap polymer (PTB7-Th) for high performance. Adv. Mater..

[86] Mühlbacher D., Scharber M., Morana M., Zhu Z., Waller D., Gaudiana R., Brabec C. (2006). High photovoltaic performance of a low-bandgap polymer. Adv. Mater..

[87] Soci C., Hwang I.W., Moses D., Zhu Z., Waller D., Gaudiana R., Brabec C.J., Heeger A.J. (2007). Photoconductivity of a low-bandgap conjugated polymer. Adv. Funct. Mater..

[88] Huo L., Liu T., Sun X., Cai Y., Heeger A.J., Sun Y. (2015). Single-junction organic solar cells based on a novel wide-bandgap polymer with efficiency of 9.7%. Adv. Mater..

[89] Qian D., Ye L., Zhang M., Liang Y., Li L., Huang Y., Guo X., Zhang S., Tan Z.A., Hou J. (2012). Design, application, and morphology study of a new photovoltaic polymer with strong aggregation in solution state. Macromolecules.

[90] Yuan J., Huang T., Cheng P., Zou Y., Zhang H., Yang J.L., Chang S.-Y., Zhang Z., Huang W., Wang R. (2019). Enabling low voltage losses and high photocurrent in fullerene-free organic photovoltaics. Nat. Commun..

[91] Cui Y., Yao H., Hong L., Zhang T., Xu Y., Xian K., Gao B., Qin J., Zhang J., Wei Z. (2019). Achieving over 15% efficiency in organic photovoltaic cells via copolymer design. Adv. Mater..

[92] Min J., Zhang Z.-G., Zhang S., Li Y. (2012). Conjugated side-chain-isolated D–A copolymers based on benzo[1,2-b:4,5-b′]dithiophene-alt-dithienylbenzotriazole: Synthesis and photovoltaic properties. Chem. Mater..

[93] Gao L., Zhang Z.-G., Bin H., Xue L., Yang Y., Wang C., Liu F., Russell T.P., Li Y. (2016). High-efficiency nonfullerene polymer solar cells with medium bandgap polymer donor and narrow bandgap organic semiconductor acceptor. Adv. Mater..

[94] Bin H., Zhang Z.-G., Gao L., Chen S., Zhong L., Xue L., Yang C., Li Y. (2016). Non-fullerene polymer solar cells based on alkylthio and fluorine substituted 2D-conjugated polymers reach 9.5% efficiency. J. Am. Chem. Soc..

[95] Bin H., Zhong L., Yang Y., Gao L., Huang H., Sun C., Li X., Xue L., Zhang Z.-G., Zhang Z. (2017). Medium bandgap polymer donor based on Bi(trialkylsilylthienyl-benzo[1,2-b:4,5-b′]-difuran) for high performance nonfullerene polymer solar cells. Adv. Energy Mater..

[96] Xue L., Yang Y., Xu J., Zhang C., Bin H., Zhang Z.-G., Qiu B., Li X., Sun C., Gao L. (2017). Side chain engineering on medium bandgap copolymers to suppress triplet formation for high-efficiency polymer solar cells. Adv. Mater..

[97] Bin H., Gao L., Zhang Z.-G., Yang Y., Zhang Y., Zhang C., Chen S., Xue L., Yang C., Xiao M. (2016). 11.4% Efficiency non-fullerene polymer solar cells with trialkylsilyl substituted 2D-conjugated polymer as donor. Nat. Commun..

[98] Xiong J., Jin K., Jiang Y., Qin J., Wang T., Liu J., Liu Q., Peng H., Li X., Sun A. (2019). Thiolactone copolymer donor gifts organic solar cells a 16.72% efficiency. Sci. Bull..

[99] Jiang K., Wei Q., Lai J.Y.L., Peng Z., Kim H.K., Yuan J., Ye L., Ade H., Zou Y., Yan H. (2019). Alkyl chain tuning of small molecule acceptors for efficient organic solar cells. Joule.

[100] Jin K., Xiao Z., Ding L. (2021). D18, an eximious solar polymer!. J. Semicond..

[101] Qin J., Zhang L., Zuo C., Xiao Z., Yuan Y., Yang S., Hao F., Cheng M., Sun K., Bao Q. (2021). A chlorinated copolymer donor demonstrates a 18.13% power conversion efficiency. J. Semicond..

[102] Xu X., Bi Z., Ma W., Wang Z., Choy W.C.H., Wu W., Zhang G., Li Y., Peng Q. (2017). Highly efficient ternary-blend polymer solar cells enabled by a nonfullerene acceptor and two polymer donors with a broad composition tolerance. Adv. Mater..

[103] Chen Y., Ye P., Jia X., Gu W., Xu X., Wu X., Wu J., Liu F., Zhu Z.-G., Huang H. (2017). Tuning Voc for high performance organic ternary solar cells with non-fullerene acceptor alloys. J. Mater. Chem. A.

[104] Chen Y., Ye P., Zhu Z.-G., Wang X., Yang L., Xu X., Wu X., Dong T., Zhang H., Hou J. (2017). Achieving high-performance ternary organic solar cells through tuning acceptor alloy. Adv. Mater..

[105] Lu H., Zhang J., Chen J., Liu Q., Gong X., Feng S., Xu X., Ma W., Bo Z. (2016). Ternary-blend polymer solar cells combining fullerene and nonfullerene acceptors to synergistically boost the photovoltaic performance. Adv. Mater..

[106] Fan B., Zhong W., Jiang X.-F., Yin Q., Ying L., Huang F., Cao Y. (2017). Improved performance of ternary polymer solar cells based on a nonfullerene electron cascade acceptor. Adv. Energy Mater..

[107] Bi P., Hao X. (2019). Versatile ternary approach for novel organic solar cells: A review. Sol. RRL.

[108] Huang J.-S., Goh T., Li X., Sfeir M.Y., Bielinski E.A., Tomasulo S., Lee M.L., Hazari N., Taylor A.D. (2013). Polymer bulk heterojunction solar cells employing Förster resonance energy transfer. Nat. Photonics.

[109] Lu L., Chen W., Xu T., Yu L. (2015). High-performance ternary blend polymer solar cells involving both energy transfer and hole relay processes. Nat. Commun..

[110] Zhao F., Li Y., Wang Z., Yang Y., Wang Z., He G., Zhang J., Jiang L., Wang T., Wei Z. (2017). Combining energy transfer and optimized morphology for highly efficient ternary polymer solar cells. Adv. Energy Mater..

[111] Du X., Lu X., Zhao J., Zhang Y., Li X., Lin H., Zheng C., Tao S. (2019). Hydrogen bond induced green solvent processed high performance ternary organic solar cells with good tolerance on film thickness and blend ratios. Adv. Funct. Mater..

[112] Zhong L., Gao L., Bin H., Hu Q., Zhang Z.-G., Liu F., Russell T.P., Zhang Z., Li Y. (2017). High efficiency ternary nonfullerene polymer solar cells with two polymer donors and an organic semiconductor acceptor. Adv. Energy Mater..

[113] Nian L., Kan Y., Wang H., Gao K., Xu B., Rong Q., Wang R., Wang J., Liu F., Chen J. (2018). Ternary non-fullerene polymer solar cells with 13.51% efficiency and a record-high fill factor of 78.13%. Energy Environ. Sci..

[114] Song X., Gasparini N., Nahid M.M., Paleti S.H.K., Wang J.-L., Ade H., Baran D. (2019). Dual sensitizer and processing-aid behavior of donor enables efficient ternary organic solar cells. Joule.

[115] Xu X., Feng K., Lee Y.W., Woo H.Y., Zhang G., Peng Q. (2020). Subtle polymer donor and molecular acceptor design enable efficient polymer solar cells with a very small energy loss. Adv. Funct. Mater..

[116] Xie G., Zhang Z., Su Z., Zhang X., Zhang J. (2020). 16.5% efficiency ternary organic photovoltaics with two polymer donors by optimizing molecular arrangement and phase separation. Nano Energy.

[117] Han J., Wang X., Huang D., Yang C., Yang R., Bao X. (2020). Employing asymmetrical thieno[3,4-d]pyridazin-1(2H)-one block enables efficient ternary polymer solar cells with improved light-harvesting and morphological properties. Macromolecules.

[118] An Q., Wang J., Ma X., Gao J., Hu Z., Liu B., Sun H., Guo X., Zhang X., Zhang F. (2020). Two compatible polymer donors contribute synergistically for ternary organic solar cells with 17.53% efficiency. Energy Environ. Sci..

[119] Zhou Z., Xu S., Song J., Jin Y., Yue Q., Qian Y., Liu F., Zhang F., Zhu X. (2018). High-efficiency small-molecule ternary solar cells with a hierarchical morphology enabled by synergizing fullerene and non-fullerene acceptors. Nat. Energy.

[120] Liu T., Luo Z., Fan Q., Zhang G., Zhang L., Gao W., Guo X., Ma W., Zhang M., Yang C. (2018). Use of two structurally similar small molecular acceptors enabling ternary organic solar cells with high efficiencies and fill factors. Energy Environ. Sci..

[121] Li K., Wu Y., Tang Y., Pan M.-A., Ma W., Fu H., Zhan C., Yao J. (2019). Ternary blended fullerene-free polymer solar cells with 16.5% efficiency enabled with a higher-LUMO-level acceptor to improve film morphology. Adv. Energy Mater..

[122] Zhan L., Li S., Lau T.-K., Cui Y., Lu X., Shi M., Li C.-Z., Li H., Hou J., Chen H. (2020). Over 17% efficiency ternary organic solar cells enabled by two non-fullerene acceptors working in an alloy-like model. Energy Environ. Sci..

[123] Zhu C., Yuan J., Cai F., Meng L., Zhang H., Chen H., Li J., Qiu B., Peng H., Chen S. (2020). Tuning the electron-deficient core of a non-fullerene acceptor to achieve over 17% efficiency in a single-junction organic solar cell. Energy Environ. Sci..

[124] Sun Y., Li Y., Cai Y., Xie Y., Song J., Wu H., Tang Z., Zhang J., Huang F. (2021). A facile strategy for third component selection in non-fullerene acceptor based ternary organic solar cells. Energy Environ. Sci..

[125] Cai Y., Li Y., Wang R., Wu H., Chen Z., Zhang J., Ma Z., Hao X., Zhao Y., Zhang C. (2021). A well-mixed phase formed by two compatible non-fullerene acceptors enables ternary organic solar cells with efficiency over 18.6%. Adv. Mater..

[126] Chen Z., Song W., Yu K., Ge J., Zhang J., Xie L., Peng R., Ge Z. (2021). Small-molecular donor guest achieves rigid 18.5% and flexible 15.9% efficiency organic photovoltaic via fine-tuning microstructure morphology. Joule.

[127] Jin K., Xiao Z., Ding L. (2021). 18.69% PCE from organic solar cells. J. Semicond..

[128] Koppe M., Egelhaaf H.-J., Dennler G., Scharber M.C., Brabec C.J., Schilinsky P., Hoth C.N. (2010). Near IR sensitization of organic bulk heterojunction solar cells: Towards optimization of the spectral response of organic solar cells. Adv. Funct. Mater..

[129] Yan D., Xin J., Li W., Liu S., Wu H., Ma W., Yao J., Zhan C. (2019). 13%-Efficiency quaternary polymer solar cell with nonfullerene and fullerene as mixed electron acceptor materials. ACS Appl. Mater. Interfaces.

[130] Bi Z., Zhu Q., Xu X., Naveed H.B., Sui X., Xin J., Zhang L., Li T., Zhou K., Liu X. (2019). Efficient quaternary organic solar cells with parallel-alloy morphology. Adv. Funct. Mater..

[131] Goh T., Huang J.-S., Yager K.G., Sfeir M.Y., Nam C.-Y., Tong X., Guard L.M., Melvin P.R., Antonio F., Bartolome B.G. (2016). Quaternary organic solar cells enhanced by cocrystalline squaraines with power conversion efficiencies >10%. Adv. Energy Mater..

[132] Vos A.D. (1980). Detailed balance limit of the efficiency of tandem solar cells. J. Phys. D Appl. Phys..

[133] Liu Y., Chen C.-C., Hong Z., Gao J., Yang Y., Zhou H., Dou L., Li G., Yang Y. (2013). Solution-processed small-molecule solar cells: Breaking the 10% power conversion efficiency. Sci. Rep..

[134] You J., Dou L., Yoshimura K., Kato T., Ohya K., Moriarty T., Emery K., Chen C.-C., Gao J., Li G. (2013). A polymer tandem solar cell with 10.6% power conversion efficiency. Nat. Commun..

[135] Li M., Gao K., Wan X., Zhang Q., Kan B., Xia R., Liu F., Yang X., Feng H., Ni W. (2017). Solution-processed organic tandem solar cells with power conversion efficiencies >12%. Nat. Photonics.

[136] Cui Y., Yao H., Gao B., Qin Y., Zhang S., Yang B., He C., Xu B., Hou J. (2017). Fine-tuned photoactive and interconnection layers for achieving over 13% efficiency in a fullerene-free tandem organic solar cell. J. Am. Chem. Soc..

[137] Zhang Y., Kan B., Sun Y., Wang Y., Xia R., Ke X., Yi Y.-Q.-Q., Li C., Yip H.-L., Wan X. (2018). Nonfullerene tandem organic solar cells with high performance of 14.11%. Adv. Mater..

[138] Ho C.H.Y., Kim T., Xiong Y., Firdaus Y., Yi X., Dong Q., Rech J.J., Gadisa A., Booth R., O’Connor B.T. (2020). High-performance tandem organic solar cells using HSolar as the interconnecting layer. Adv. Energy Mater..

[139] Qin S., Jia Z., Meng L., Zhu C., Lai W., Zhang J., Huang W., Sun C., Qiu B., Li Y. (2021). Non-halogenated-solvent processed and additive-free tandem organic solar cell with efficiency reaching 16.67%. Adv. Funct. Mater..

[140] Liu G., Jia J., Zhang K., Jia X.E., Yin Q., Zhong W., Li L., Huang F., Cao Y. (2019). 15% Efficiency tandem organic solar cell based on a novel highly efficient wide-bandgap nonfullerene acceptor with low energy loss. Adv. Energy Mater..

[141] Huang X., Sun B., Li Y., Jiang C., Fan D., Fan J., Forrest S.R. (2020). 15.9% organic tandem solar cell with extended near-infrared absorption. Appl. Phys. Lett..

[142] Jia Z., Qin S., Meng L., Ma Q., Angunawela I., Zhang J., Li X., He Y., Lai W., Li N. (2021). High performance tandem organic solar cells via a strongly infrared-absorbing narrow bandgap acceptor. Nat. Commun..

[143] Meng L., Zhang Y., Wan X., Li C., Zhang X., Wang Y., Ke X., Xiao Z., Ding L., Xia R. (2018). Organic and solution-processed tandem solar cells with 17.3% efficiency. Science.

[144] Liu G., Xia R., Huang Q., Zhang K., Hu Z., Jia T., Liu X., Yip H.-L., Huang F. (2021). Tandem organic solar cells with 18.7% efficiency enabled by suppressing the charge recombination in front sub-cell. Adv. Funct. Mater..

[145] Salim M.B., Nekovei R., Jeyakumar R. (2020). Organic tandem solar cells with 18.6% efficiency. Sol. Energy.

[146] Moiz S.A., Nahhas A.M., Um H.-D., Jee S.-W., Cho H.K., Kim S.-W., Lee J.-H. (2012). A stamped PEDOT:PSS–silicon nanowire hybrid solar cell. Nanotechnology.

[147] Moiz S.A., Alahmadi A.N.M., Aljohani A.J. (2020). Design of silicon nanowire array for PEDOT:PSS-silicon nanowire-based hybrid solar cell. Energies.

[148] Kim M.S., Lee J.H., Kwak M.K. (2020). Review: Surface texturing methods for solar cell efficiency enhancement. Int. J. Precis. Eng. Manuf..

[149] Zhao F., Wang C., Zhan X. (2018). Morphology control in organic solar cells. Adv. Energy Mater..

[150] Zhang H., Li Y., Zhang X., Zhang Y., Zhou H. (2020). Role of interface properties in organic solar cells: From substrate engineering to bulk-heterojunction interfacial morphology. Mater. Chem. Front..

[151] Speller E.M., Clarke A.J., Luke J., Lee H.K.H., Durrant J.R., Li N., Wang T., Wong H.C., Kim J.-S., Tsoi W.C. (2019). From fullerene acceptors to non-fullerene acceptors: Prospects and challenges in the stability of organic solar cells. J. Mater. Chem. A.

[152] Duan L., Uddin A. (2020). Progress in stability of organic solar cells. Adv. Sci..

[153] Doumon N.Y., Dryzhov M.V., Houard F.V., Le Corre V.M., Rahimi Chatri A., Christodoulis P., Koster L.J.A. (2019). Photostability of fullerene and non-fullerene polymer solar cells: The role of the acceptor. ACS Appl. Mater. Interfaces.

[154] Gu Y., Liu Y., Russell T.P. (2020). Fullerene-based interlayers for breaking energy barriers in organic solar cells. ChemPlusChem.

[155] Halls J.J.M., Pichler K., Friend R.H., Moratti S.C., Holmes A.B. (1996). Exciton diffusion and dissociation in a poly(p-phenylenevinylene)/C60 heterojunction photovoltaic cell. Appl. Phys. Lett..

[156] Larsen C., Lundberg P., Tang S., Ràfols-Ribé J., Sandström A., Mattias Lindh E., Wang J., Edman L. (2021). A tool for identifying green solvents for printed electronics. Nat. Commun..

[157] Farahat M.E., Laventure A., Anderson M.A., Mainville M., Tintori F., Leclerc M., Ratcliff E.L., Welch G.C. (2020). Slot-die-coated ternary organic photovoltaics for indoor light recycling. ACS Appl. Mater. Interfaces.

